# Tree polynomials identify a link between co-transcriptional R-loops and nascent RNA folding

**DOI:** 10.1371/journal.pcbi.1012669

**Published:** 2024-12-13

**Authors:** Pengyu Liu, Jacob Lusk, Nataša Jonoska, Mariel Vázquez

**Affiliations:** 1 Department of Microbiology and Molecular Genetics, University of California, Davis, Davis, California, United States of America; 2 Department of Mathematics and Statistics, University of South Florida, Tampa, Florida, United States of America; 3 Department of Mathematics, University of California, Davis, Davis, California, United States of America; University of Missouri, UNITED STATES OF AMERICA

## Abstract

R-loops are a class of non-canonical nucleic acid structures that typically form during transcription when the nascent RNA hybridizes the DNA template strand, leaving the non-template DNA strand unpaired. These structures are abundant in nature and play important physiological and pathological roles. Recent research shows that DNA sequence and topology affect R-loops, yet it remains unclear how these and other factors contribute to R-loop formation. In this work, we investigate the link between nascent RNA folding and the formation of R-loops. We introduce tree-polynomials, a new class of representations of RNA secondary structures. A tree-polynomial representation consists of a rooted tree associated with an RNA secondary structure together with a polynomial that is uniquely identified with the rooted tree. Tree-polynomials enable accurate, interpretable and efficient data analysis of RNA secondary structures without pseudoknots. We develop a computational pipeline for investigating and predicting R-loop formation from a genomic sequence. The pipeline obtains nascent RNA secondary structures from a co-transcriptional RNA folding software, and computes the tree-polynomial representations of the structures. By applying this pipeline to plasmid sequences that contain R-loop forming genes, we establish a strong correlation between the coefficient sums of tree-polynomials and the experimental probability of R-loop formation. Such strong correlation indicates that the pipeline can be used for accurate R-loop prediction. Furthermore, the interpretability of tree-polynomials allows us to characterize the features of RNA secondary structure associated with R-loop formation. In particular, we identify that branches with short stems separated by bulges and interior loops are associated with R-loops.

## Author summary

R-loops are associated with various regulatory processes and with genome instability and diseases such as cancer and genetic disorders. To date, understanding the mechanisms of R-loop formation and predicting the occurrence of R-loops remain important and timely tasks. R-loops form during transcription when the nascent RNA hybridizes with the DNA template strand. Within this process, the R-loop competes with the nascent RNA self-folding secondary structure. Here, we show a possible link between R-loop formation and nascent RNA secondary structures. First we introduce a novel representation of RNA secondary structures using a class of mathematical objects called tree-polynomials. Next we develop a computational pipeline to predict R-loop formation. Through this pipeline, we identify a strong link between nascent RNA secondary structures and R-loop formation. Furthermore, tree-polynomials enable the characterization of RNA secondary structures associated with R-loops. These results suggest a potential mechanism of R-loop formation as a result of nascent RNA folding structures.

## Introduction

R-loops are three-stranded nucleic acid structures abundant in the genomes of bacteria, plants and mammals, that play important roles in physiological and pathological processes [1–4]. The mechanisms of R-loop formation and the factors that drive their occurrence remain unclear. Studies involving genome mapping, biochemical reconstitution and energy-based mathematical models indicate that DNA sequence and topology strongly influence R-loop formation [1]. In particular, R-loops correlate with GC-rich and CG-skew sequences on the template strand as well as with negative supercoiling [5, 6]. Co-transcriptional R-loops form during transcription when the nascent RNA hybridizes with the DNA template, leaving the unpaired DNA coding strand free to wrap around the RNA:DNA duplex or to adopt a non-standard structure [7–9]. In this work, we show a potential link between the secondary structure of co-transcriptional RNA and the R-loop formation.

**Fig 1:**
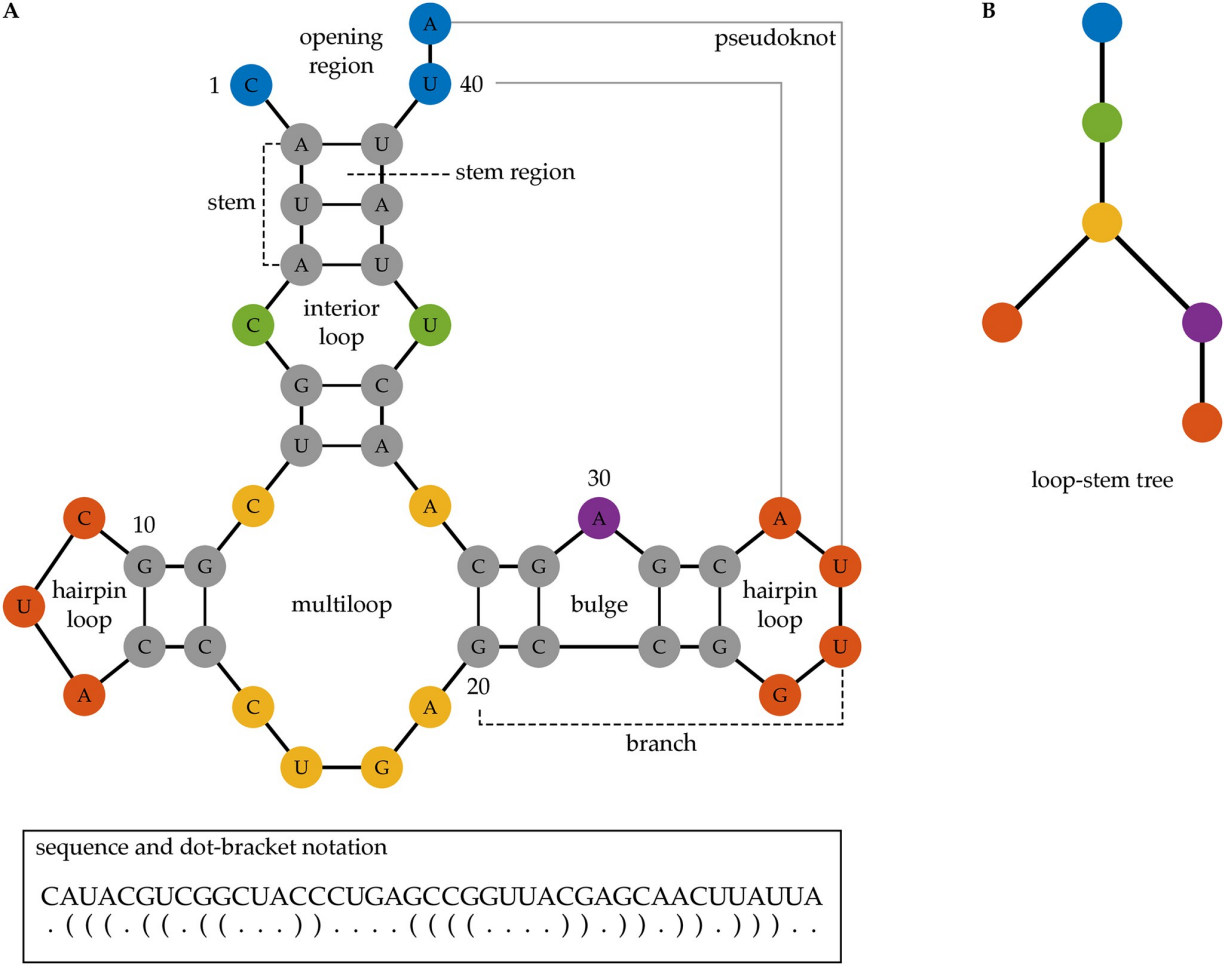
RNA secondary structure, dot-bracket notation and rooted tree representation. Panel A shows the secondary structure of a 41 nucleotides (nt) long RNA molecule. Each node represents a nucleotide (A, U, G, or C). The colors indicate different regions of the RNA secondary structure. Integers indicate the nucleotide position along the RNA sequence. Solid black edges represent covalent or hydrogen bonds, and gray edges represent bonds between distant nucleotides forming a pseudoknot. The bottom insert shows the sequence of the RNA molecule and the dot-bracket notation. Panel B shows the loop-stem tree representation of the RNA secondary structure in panel A without the pseudoknot. Here, every vertex represents a loop, and the colors of the vertices correspond to different regions. The blue vertex representing the opening region is the root of the tree. Every edge in the tree represents a stem of the RNA secondary structure.

The secondary structure of an RNA molecule can be described by its paired and unpaired nucleotides (Fig 1A). The paired nucleotides form double-stranded helices that are interspersed with unpaired nucleotides that form loops or bubbles [10]. We can represent an RNA secondary structure with a graph, a mathematical object consisting of vertices (nodes) connected by edges (Fig 1B) [11]. Rooted trees are graphs that provide a simple and efficient way to capture essential features of RNA secondary structures without pseudoknots [11, 12]. The use of rooted trees opens the door to a wealth of efficient graph theoretical methods for designing, comparing and analyzing RNA secondary structures [13–15].

Graph polynomials are powerful mathematical objects that capture important information of discrete structures, with the Tutte polynomial [16] being a notable example. Graph polynomials, encoded as coefficient vectors or matrices, have the advantage of being compatible with data analytic tools, including distance-based and machine learning methods. In [17], the author introduced a method that assigns every unlabeled tree a unique 2-variable polynomial representation; we refer to it as *polynomial P*. Polynomial *P* has been successfully applied to analyze pathogen evolution [18] and syntax of languages [19].

**Fig 2:**
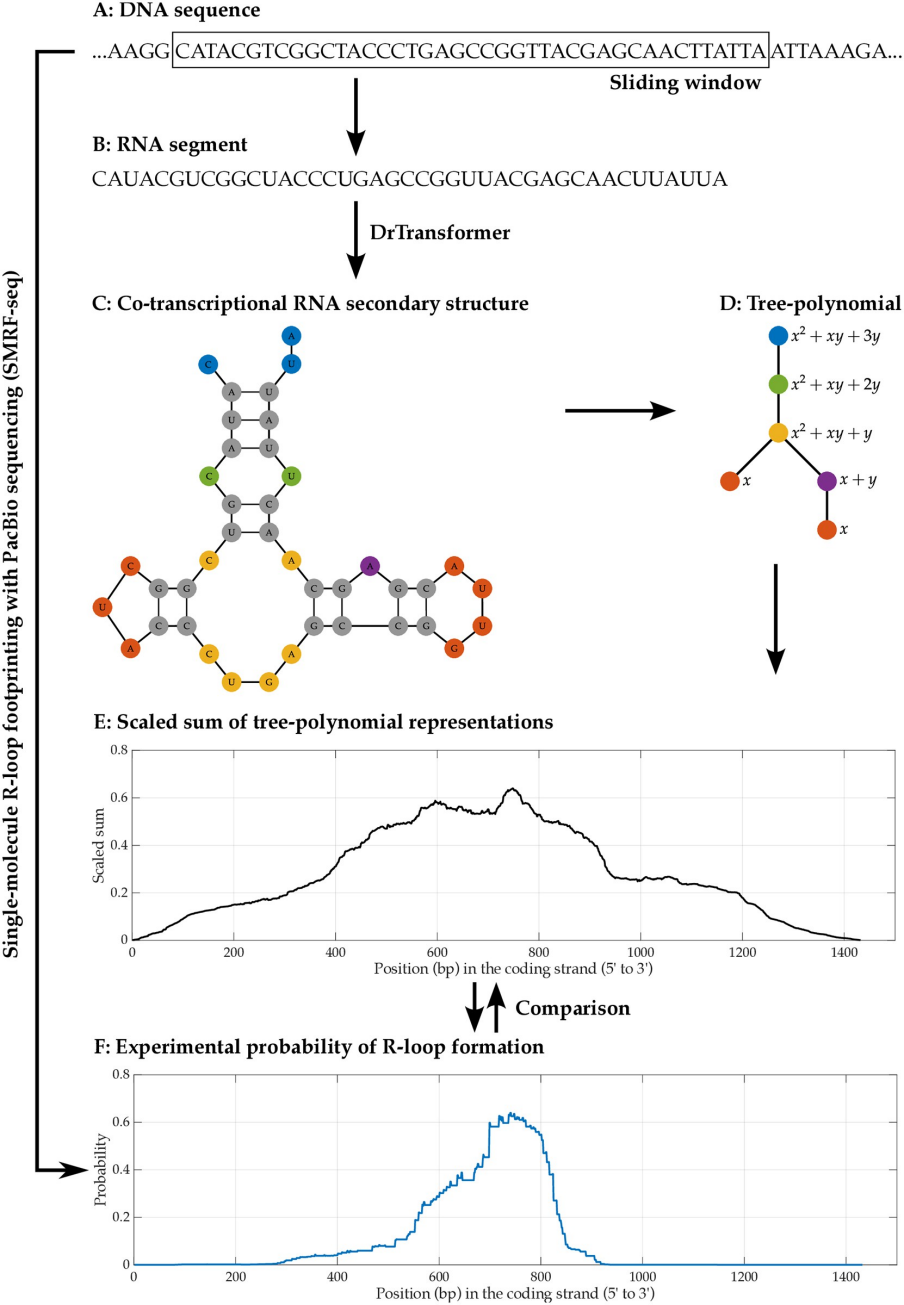
The computational pipeline for investigating and predicting R-loop formation. This figure shows a brief pictorial description of the computational pipeline for predicting R-loop formation using DrTransformer and tree-polynomial. This pipeline takes a DNA sequence (panel A) as an input and covert the DNA sequence into the corresponding RNA sequence (panel B). Then, we use DrTransformer to predict co-transcriptional RNA secondary structures (panel C) of the RNA sequence. We use tree-polynomials (panel D) to represent the secondary structures obtained from DrTransformer, and compute the scaled sums of the tree-polynomials (panel E). To test the performance of the pipeline in predicting R-loop formation, we compare the scaled sums to the experimental probabilities of R-loop formation (panel F) obtained using SMRF-seq. See S8 Fig for more detail.

**Fig 3:**
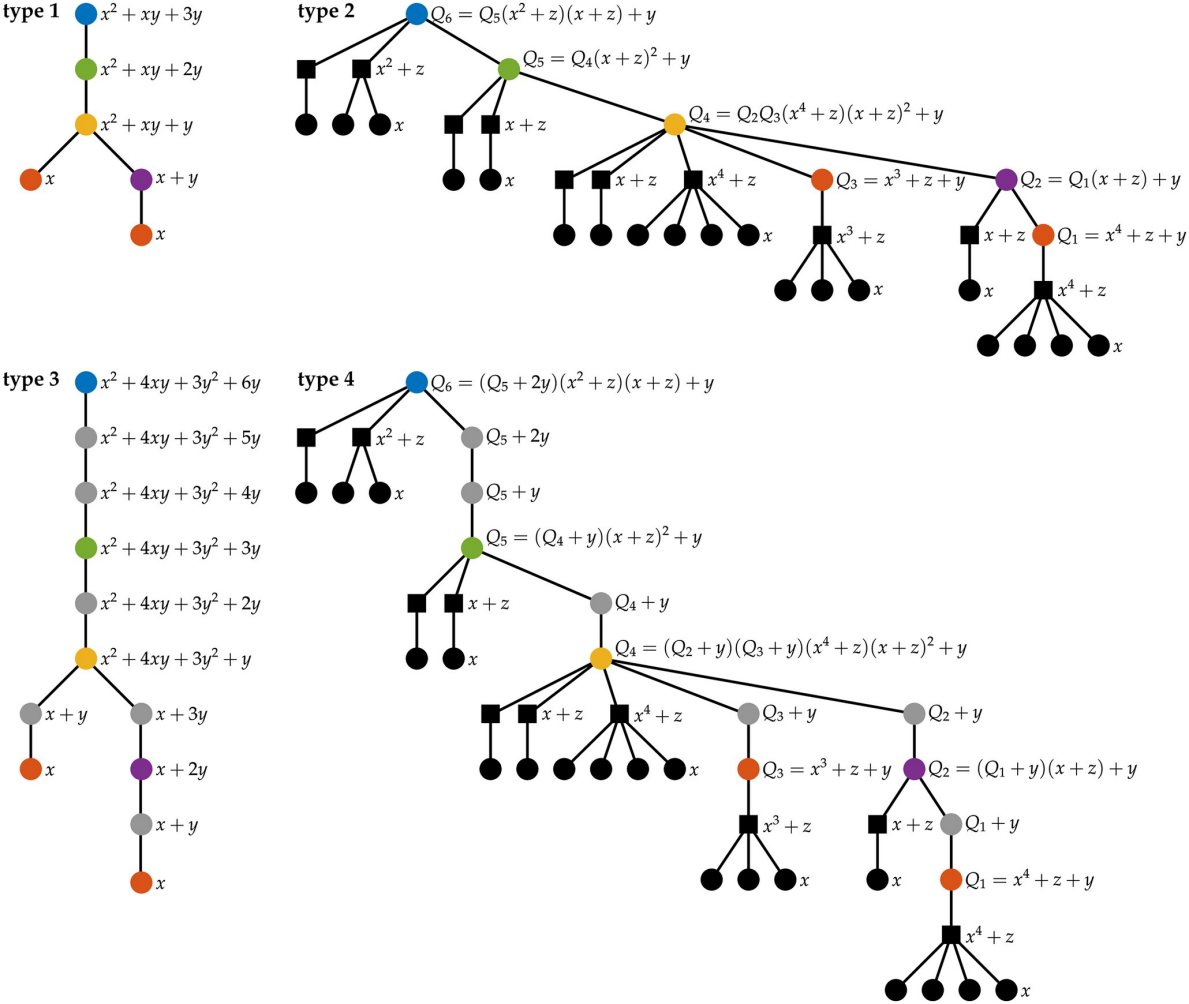
Tree-polynomial representations of an RNA secondary structure. The figure shows the first four types of tree-polynomial representations of the RNA secondary structure in Fig 1. Each tree-polynomial representation of an RNA secondary structure consists of a rooted tree representation and its corresponding polynomial. Vertices in the trees have the same colors as the loops and stem regions that they represent. The black round vertices represent unpaired nucleotides. The black square vertices in the type 2 and the type 4 tree representations indicate artificial internal vertices introduced to group unpaired nucleotides. In each rooted tree representation, we illustrate the recursive process of computing the corresponding polynomial from leaf vertices to the root vertex. The polynomial at the root vertex (blue) of a tree is the polynomial that represents the tree. Polynomial *P* is associated with type 1 and type 3 trees, and polynomial *Q* is associated with type 2 and type 4 trees.

In this study, we introduce *tree-polynomials*, a new class of RNA secondary structure representations. Tree-polynomials are a useful tool that can characterize and reveal structural properties. We define a tree-polynomial representation of an RNA secondary structure without pseudoknots to be a rooted tree associated with the structure together with a polynomial uniquely identifying the tree (Figs 1, 2 and 3). Here, we define new rooted tree representations for RNA secondary structures and generalize the polynomial *P* to a new 3-variable polynomial *Q*. These new mathematical objects allow us to better capture structural features of an RNA secondary structure. Tree-polynomials enable accurate, comprehensive, interpretable and easy-to-compute data analysis of RNA secondary structures. We validate our approach by applying tree-polynomials to cluster secondary structures of non-coding RNAs (ncRNAs) obtained from the bpRNA-1m database [20] showing that tree-polynomials can distinguish different families of ncRNA secondary structures.

We use tree-polynomials to test the hypothesis that there is a link between the co-transcriptional RNA secondary structure and R-loop formation. We develop a computational pipeline to investigate and predict R-loop formation from a genomic sequence (Fig 2). The pipeline uses DrTransformer, a recently developed co-transcriptional RNA folding model [21], to obtain nascent RNA secondary structures and compute tree-polynomials of the structures. We apply the pipeline to two plasmids that contain R-loop forming genes [6] with available experimental data obtained by single-molecule R-loop footprinting and sequencing (SMRF-seq). We find the coefficient sums of the tree-polynomials highly correlate with the experimental probabilities of R-loop formation. This strong correlation indicates the usefulness of this approach for accurate R-loop prediction. In addition, the interpretability of the tree-polynomials allows us to characterize RNA secondary structures associated with R-loop formation. We identify that RNA secondary structures whose tree-polynomials have large coefficient sums have many linear branches with multiple short stems separated by bulges and interior loops. These structural features with non-attached branched nucleotides potentially increase the chance of initiating strand migration between the nascent RNA and the DNA template strand, hence increasing the probability of R-loop formation [22, 23].

## Results

### Tree-polynomial representations of RNA secondary structures

#### Rooted tree representations recording structural features

We study four structural features of an RNA secondary structure (without pseudoknots) and define rooted tree representations that capture all combinations of their appearances. The *loop-stem relation* feature describes how loops and stems are connected in an RNA secondary structure; they can be represented by the loop-stem tree (Fig 1). This is the base for all other trees in the tree-polynomial representations. The vertices are the loops and the edges indicate the stems that connect the loops. The root of this tree represents the 5′ and 3′ end of the RNA. The *loop size* feature describes the number of unpaired nucleotides around each loop. For example, the loop size of the RNA secondary structure in Fig 1A is 3 for the opening region, 2 for the interior loop, 6 for the multiloop, 3 for the left hairpin loop, 1 for the bulge and 4 for the right hairpin loop. The *stem size* feature describes the number of base pairs in each stem. The RNA secondary structure in Fig 1A has one stem with 3 base pairs (bp) and four stems with 2bp. The *loop group* feature describes how the stems around a loop separate the unpaired nucleotides into groups. In Fig 1A, the opening region has two loop groups with 1 and 2 unpaired nucleotides, respectively. The interior loop has two loop groups with 1 nucleotide (nt), and the multiloop has three loop groups with 1, 4, and 1nt, respectively. Every bulge and hairpin loop has only one loop group. See Materials and methods for more details.

The loop size, stem size and loop group are quantitative features that combine with the loop-stem relation to construct rooted tree representations of RNA secondary structures. Every rooted tree representation contains the loop-stem relation. There are eight possible ways to record each of the other three features (loop size, stem size and loop group). *Type 1* trees record only the loop-stem relation, called *loop-stem trees* [11], and is part of all eight types of trees. We show the features represented by each of the eight types of trees in Table 1. We note that type 6 trees are the arc trees defined in [12]. Fig 3 shows the type 1, 2, 3 and 4 trees for the RNA secondary structure in Fig 1A, and S1 Fig shows its type 5, 6, 7 and 8 trees.

**Table 1:** Definition and performance of the eight tree-polynomial representations of RNA secondary structures.

Tree type	Poly-nomial	Loop-stem	Loop size	Stem size	Loop group	MMR	PCC pFC8	PCC pFC53
1^†^	P	✓				6.82%	0.77	0.79
2	Q	✓	✓		✓	17.07%	0.96	0.96
3	P	✓		✓		3.40%*	-0.30	-0.19
4	Q	✓	✓	✓	✓	17.51%	0.97*	0.97*
5	P	✓	✓			9.29%	0.60	0.64
6^†^	P	✓	✓	✓		9.70%	-0.31	-0.21
7	P	✓			✓	24.32%	0.60	0.64
8	P	✓		✓	✓	22.16%	-0.31	-0.21

The first two columns show the types of tree-polynomial representations. Obelisks (†) indicate previously studied tree representations [11, 12]. The middle four columns show the features (loop-stem relation, loop size, stem size and loop group) captured by each tree-polynomial representation. The last three columns show the mean misclassification rates (MMR) in clustering the 735 ncRNA secondary structures in the bpRNA-Rfam-7 dataset (see Materials and methods) and the Pearson’s correlation coefficients (PCC) between the scaled sums (with the highest PCC in the last 10 transcription steps) and the experimental R-loop formation probabilities of supercoiled pFC8 and pFC53 plasmid [6] (see Data and Experiments section), respectively. Asterisks indicate the top performing tree-polynomial representations in each of these experiments.

The newly defined type 2 and 4 trees that record the loop group and the loop size features can differentiate RNA secondary structures that are indistinguishable by the other six tree representations (S2 Fig). Type 7 and 8 tree representations that record the loop group feature but not the loop size feature provide little additional information about RNA secondary structures compared to the other six tree representations. S1 Appendix contains more details about the eight tree representations.

#### Tree-polynomial representations

A *tree-polynomial representation*, or a *tree-polynomial* for short, of an RNA secondary structure consists of a rooted tree representation and a tree-distinguishing polynomial, such as the 2-variable polynomial *P* [17]. The polynomial *P* can be applied to any tree representation type to define a tree-polynomial. Fig 3 illustrates the polynomial *P* for the type 1 and 3 trees as well as the recursive calculation that gives rise to it. Polynomial *P* is interpretable. For example, given a type 1 (or type 3) tree *T*, the term *x*^*n*^ in *P*(*T*, *x*, *y*) indicates that *T* has *n* leaf vertices and the RNA secondary structure has *n* hairpin loops. If *T* is a type 5 (or type 6) tree, then the term *x*^*n*^ in *P*(*T*, *x*, *y*) indicates that the RNA secondary structure has *n* unpaired nucleotides. See Materials and methods for more details.

The type 2 and 4 tree representations of an RNA secondary structure have two different classes of nodes, one class of nodes (black circles in Fig 3) corresponds to loops, stem regions and unpaired nucleotides in the structure, and the other class (black squares in Fig 3) consists of artificial internal vertices introduced to capture the loop group feature of the structure. To better distinguish these new features of the trees, we generalize the polynomial *P* to a *polynomial Q*, a 3-variable tree distinguishing polynomial.

Let *T* be the type 2 (or type 4) tree representation of an RNA secondary structure. We define the polynomial *Q*(*T*, *x*, *y*, *z*) = *Q*(*r*, *x*, *y*, *z*)recursively starting from leaf vertices to the root vertex which is denoted by *r*. Let *v* be a vertex in the tree *T*. If *v* is a leaf vertex, then *Q*(*v*, *x*, *y*, *z*) = *x*. If *v* is an artificial internal vertex (black square in Fig 3) with *k* child vertices *u*_1_, *u*_2_, …, *u*_*k*_, then $Q\left(v,x,y,z\right)=z+\Pi_{i=1}^{k}Q\left(u_{i},x,y,z\right)$. If *v* is an internal vertex that represents a loop or a stem region and has *k* child vertices *u*_1_, *u*_2_, …, *u*_*k*_, then $Q\left(v,x,y,z\right)=y+\Pi_{i=1}^{k}Q\left(u_{i},x,y,z\right)$. The polynomial *Q* for the tree *T* is the polynomial at the root vertex. Fig 3 illustrates the polynomial *Q* for the type 2 and type 4 trees as well as the recursive process for its computation. Note that substituting the variable *z* with *y* in the polynomial *Q*(*T*, *x*, *y*, *z*) of a type 2 (or type 4) tree *T* yields the polynomial *P*(*T*, *x*, *y*) for the tree *T*.

Similar to polynomial *P*, polynomial *Q* is also interpretable. The variables *x*, *y* and *z* encode structural information of the unpaired nucleotides, the loops and the groups of unpaired nucleotides around each loop, respectively. For example, given a type 2 (or type 4) tree representation *T*, the term *x*^*n*^ in the polynomial *Q*(*T*, *x*, *y*, *z*) indicates that the tree *T* has *n* leaf vertices and the RNA secondary structure has *n* unpaired nucleotides. The term *z*^*u*^ in *Q*(*T*, *x*, *y*, *z*) indicates that the RNA secondary structure has *u* groups of unpaired nucleotides in total. If we evaluate *Q* at *z* = 1, the coefficient *ℓ* in term *ℓ* *y* in *Q*(*T*, *x*, *y*, 1) indicates that the RNA secondary structure has *ℓ* loops in total. If *T* is a type 4 tree, then the RNA secondary structure has *ℓ* loops and stem regions.

We introduce eight tree-polynomials corresponding to the eight types of tree representations (Table 1). Since only trees of type 2 and 4 contain two additional classes of nodes, they are paired with polynomial *Q* to form tree-polynomial representations. Specifically, polynomial *Q* differentiates between the artificial internal vertices (indicated as black squares in Fig 3) introduced to group unpaired nucleotides and the internal vertices that represent loops or stem regions in type 2 and type 4 trees. Other tree types are paired with polynomial *P*.

#### RNA secondary structure comparison using tree-polynomial distances

We measure the similarity between RNA secondary structures, represented as tree-polynomials, by applying distance-based methods to the coefficients of the tree-polynomials [18]. Here we define the distance for polynomial *Q*. The distance for polynomial *P* is defined analogously by reducing one variable (See Materials and methods). We represent the polynomial *Q*(*T*, *x*, *y*, *z*) of degree *n* for a rooted tree *T* as an (*n* + 1) × (*n* + 1) × (*n* + 1) coefficient matrix. Each (*i*, *j*, *k*) entry of the coefficient matrix is the coefficient *c*^(*i*,*j*,*k*)^ of the term *c*^(*i*,*j*,*k*)^*x*^*i*^*y*^*j*^*z*^*k*^ in *Q*(*T*, *x*, *y*, *z*), where 0 ≤ *i*, *j*, *k* ≤ *n*. Given two type 2 (or type 4) trees *S* and *T*, let *b*^(*i*,*j*,*k*)^ and *c*^(*i*,*j*,*k*)^ be the coefficients in *Q*(*S*, *x*, *y*, *z*) and *Q*(*T*, *x*, *y*, *z*), respectively. We define the *polynomial Q distance* between *S* and *T*, *d*_*Q*_(*S*, *T*), as in Formula (1), where the function *κ* is the relative coefficient difference defined by Formula (2).

$$\begin{array}{c} d_{Q}\left(S,T\right)=\sum_{0\leq i,j,k\leq n}\kappa \left(b^{\left(i,j,k\right)},c^{\left(i,j,k\right)}\right) \end{array}$$ (1)

$$\begin{array}{c} \kappa \left(b,c\right)=\{\begin{array}{cc} |b-c|/\left(b+c\right) & \;\text{if}\;b\neq 0\;\text{or}\;c\neq 0\\ 0 & \;\text{if}\;b=0\;\text{and}\;c=0 \end{array} \end{array}$$ (2)

#### Tree-polynomial distances differentiate non-coding RNA families

To validate our approach, we apply the eight tree-polynomials and their corresponding polynomial distances to cluster the 735 secondary structures of non-coding RNAs (ncRNAs) in the bpRNA-Rfam-7 dataset [20, 24]. The bpRNA-Rfam-7 dataset contains secondary structures from seven families of ncRNAs including 5.8S ribosomal RNA and U12 minor spliceosomal RNA that play an important role in gene regulation and editing [25, 26]. We choose the bpRNA-Rfam-7 dataset because the ncRNAs have similar length and secondary structures associated with the sequences. Therefore, the bpRNA-Rfam-7 dataset is better suited for RNA secondary structure comparison. See Materials and methods, S1 Appendix, S1 and S2 Tables for more information about the bpRNA-Rfam-7 dataset.

First, we cluster the tree-polynomial representation of ncRNA secondary structures within the bpRNA-Rfam-7 dataset by applying the *k*-medoids clustering algorithm [27] using the tree-polynomial distances. Then, we compare the clustering results to the true families of the ncRNAs. In Table 1, we report the mean misclassification rate (MMR) for each of the eight tree-polynomials and their corresponding polynomial distance. The MMR measures the clustering accuracy. Within the bpRNA-Rfam-7 dataset, the type 3 tree-polynomial has the highest accuracy in clustering the ncRNA (MMR = 3.40%). The type 1 tree-polynomial has the second top performance (MMR = 6.82%). S3 and S4 Figs illustrate the pairwise tree-polynomial distances based on each tree-polynomial type.

The clustering results for the ncRNAs in the bpRNA-Rfam-7 dataset suggest that tree-polynomials that record more features of the secondary structures do not necessarily perform better. The loop-stem relation is preserved within the functional ncRNAs, and type 1 and 3 polynomials record precisely that relation. However, the number of unpaired nucleotides within the loops can vary drastically among different species, so type 2 and 4 polynomials that record loop sizes tend to misclassify those structures. This accounts for the fact that type 1 and 3 tree-polynomials perform better than type 2 and 4. See S5 and S6 Figs for examples ncRNA secondary structures in the bpRNA-Rfam-7 dataset.

To further benchmark the performance of the tree-polynomial distances, we extend our analysis to the bpRNA-Rfam-large dataset with 16,039 RNA secondary structures. The results show consistent clustering accuracy similar to those from the bpRNA-Rfam-7 dataset, as measured by MMR (S3 Table).

### RNA secondary structure and R-loop formation

#### Nascent RNA secondary structure features strongly correlate with R-loop formation

It is assumed that R-loops form behind the polymerase shortly after transcription, when the RNA hybridizes with the template DNA. R-loop formation is driven by RNA:DNA interaction and strand branch migration. Thus, the unpaired nucleotides in the RNA as the molecule exits the polymerase may initiate the branch migration. We hypothesize that there is a link between the secondary structure of the nascent RNA and the probability of R-loop formation. We develop a computational pipeline to test this hypothesis.

We use publicly available SMRF-seq experimental data of plasmid sequences that carry mammalian genes and are known to form R-loops, pFC8 and pFC53 [6]. SMRF-seq detects R-loops within a single molecule with nucleotide precision [28, 29], therefore it is suitable for analyzing the RNA secondary structures associated with specific R-loop locations. We scan each plasmid’s amplicon region with a 200nt-long sliding window. For each 200nt-long segment, we obtain 200 secondary structures, one for each transcription step using DrTransformer, an energy-based co-transcriptional RNA folding model [21]. To each RNA secondary structure we associate a type *k* (*k* = 1, 2, …, 8) tree-polynomial representation. We compute the coefficient sum of the type *k* tree-polynomial, of each obtained secondary structure and call it the *type k coefficient sum*. In this way, each RNA secondary structure corresponds to eight coefficient sums. Next, we compare the coefficient sums at each transcription step to the experimentally obtained probability of R-loop formation at that nucleotide. For each *k*, we obtain the *type k scaled sum* of a plasmid from the type *k* coefficient sums simulating the experimental probability results of R-loop formation [6, 28, 29]. See Materials and methods, S1 Appendix, S7 and S8 Figs for more details about the plasmids, the experimental data, the definitions and the process.

The scaled sums of RNA secondary structures at the last 10 transcription steps of each 200nt segment provide the most complete structural information; we focus on these structures. See S1 Appendix, S4 and S5 Tables and the interactive 3D figures available from https://github.com/Arsuaga-Vazquez-Lab/RNA-Polynomial for analogous results at other transcription steps. We compare such obtained coefficient sums at each nucleotide with the experimental probability that the nucleotide is in an R-loop. The experimental probability is obtained as the number of R-loops containing the given nucleotide divided by the total number of R-loops in the corresponding dataset. Experimentally, the R-loop probabilities were obtained from hundreds of single molecule R-loop detection readings (1993 and 626 R-loops for supercoiled pFC8 and pFC53, respectively).

**Fig 4:**
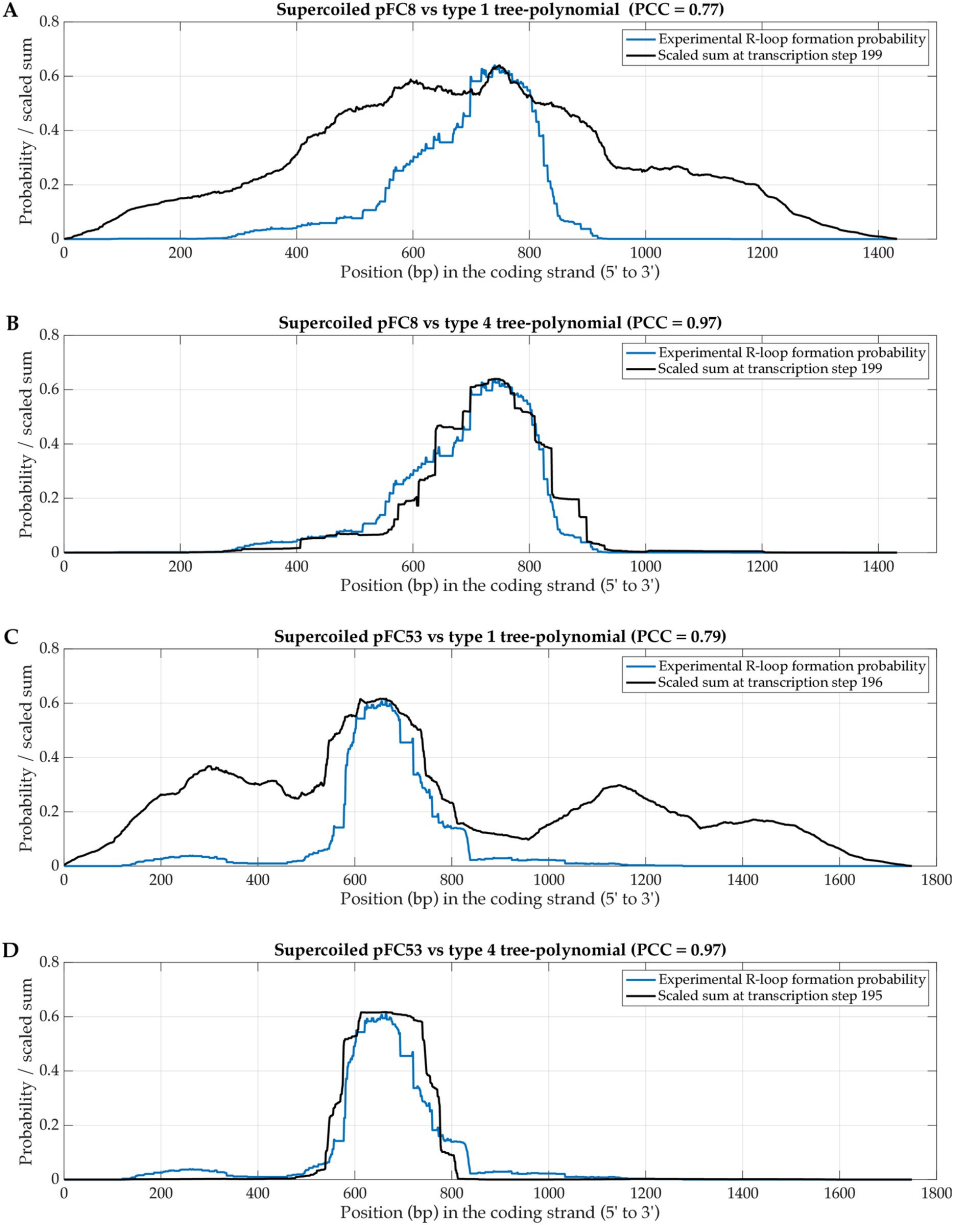
The correlations between the scaled sums and the R-loop formation probabilities of supercoiled pFC8 and pFC53 plasmids (see Data and Experiments section). The figure shows the experimental probability of R-loop formation for the supercoiled pFC8 plasmid with the type 1 (panel A) and the type 4 (panel B) scaled sums, and the experimental probability of R-loop formation for the supercoiled pFC53 plasmid with the type 1 (panel C) and the type 4 (panel D) scaled sums. The experimental probabilities of R-loop formation are from [6]. The displayed scaled sums have the highest PCC in the last ten transcription steps.

We observe a strong positive correlation between the experimental probabilities of R-loop formation and the scaled sums of tree-polynomial types 1, 2 and 4 of negatively supercoiled and hyper-negatively supercoiled pFC8 and pFC53 plasmids; see Figs 4, S9 and S10. The corresponding Pearson’s correlation coefficients (PCCs) confirm the strong correlation; see Tables 1, S4 and S5. In [6], the authors identified R-loop forming sequence clusters in the amplicon regions of pFC8 and pFC53. For each plasmid, the *major peak* refers to the sequence cluster with the highest probability of R-loop formation. The *minor peak* is the sequence cluster with the second highest probability of R-loop formation. Minor peaks are more visible for hyper-negatively supercoiled plasmids; see S9 Fig The type 2 and type 4 scaled sums closely match the probabilities of R-loop formation at the major peak with high PCCs (≥ 0.96) (Table 1 and Fig 4). Despite relatively lower PCCs (≥ 0.77), the type 1 scaled sums suggest the positions of other R-loop forming sequence clusters.

#### Branches with bubbles separating short stems drive the strong correlation

**Fig 5:**
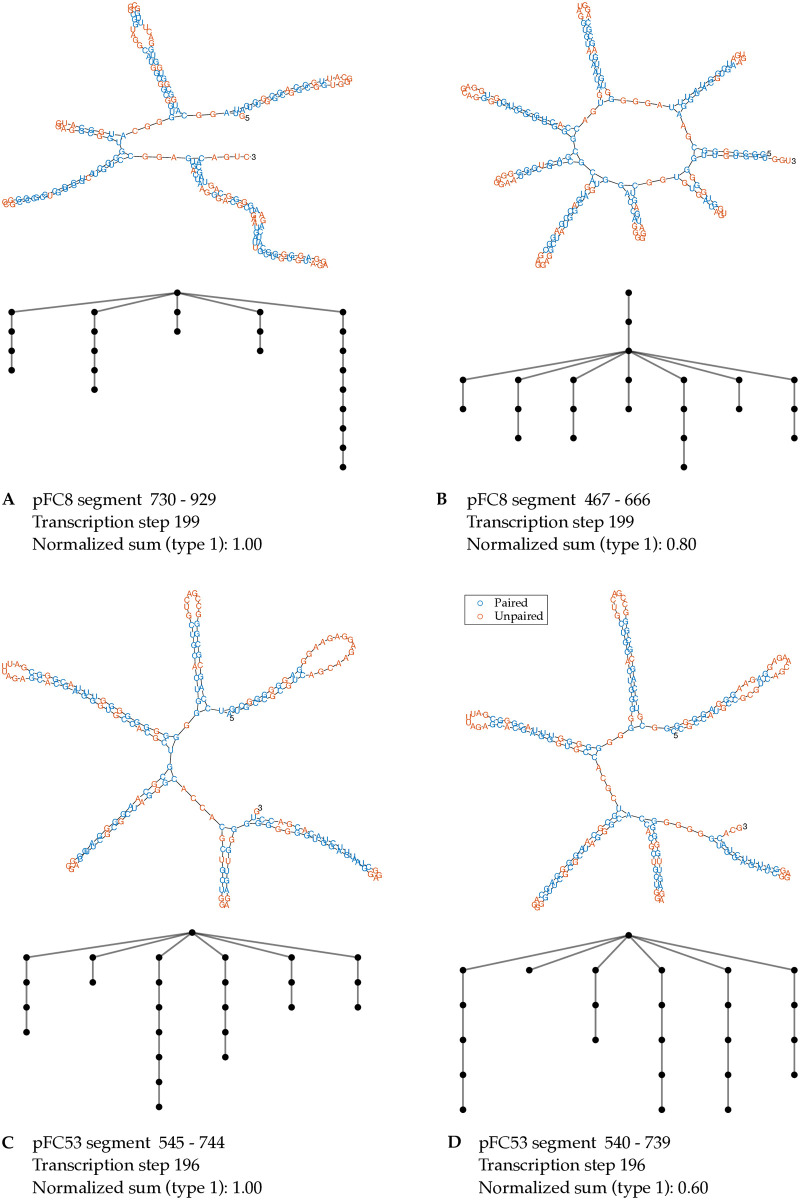
Secondary structures of RNA segments with the largest type 1 coefficient sums. The figure shows the secondary structures of RNA segments of pFC8 (panel A and B) and of pFC53 (panel C and D) with the two largest type 1 coefficient sums at the transcription step with the highest PCC in the last 10 transcription steps. Type 1 tree representations follow the corresponding secondary structures.

We investigate what features of RNA secondary structures contribute to large coefficient sums, and thus to higher probabilities of R-loop formation. Fig 5 shows the structures with the two largest type 1 coefficient sums. S11 and S12 Figs show the structures with the two largest type 2 and type 4 coefficient sums, respectively. All of these structures have linear branches with multiple bulges and interior loops separated by short stems. We refer to bulges and interior loops in a linear branch as *bubbles*. Note that a linear branch corresponds to a path in the type 1 tree representation of an RNA secondary structure such that the length of the path corresponds to the number of bubbles in the linear branch. Moreover, the vertices visited by this path have at most one child. Each path of this type contributes with a term *x* + *ℓy* where *ℓ* indicates the number of bubbles in the branch. The RNA secondary structures that correspond to large type 1 coefficient sums contain several linear branches with multiple bubbles, as observed in Fig 5. To obtain the type 1 tree-polynomial of the secondary structure we multiply the terms that correspond to these branches, which causes the coefficient sum to increase exponentially with the number of bubbles.

The arguments above imply that bulges and interior loops contribute equally to the coefficient sums of type 1 tree-polynomials. In type 2 and type 4 tree polynomials, interior loops contribute more to the coefficient sums than bulges. This is because type 2 (or type 4) trees of a linear branch contain a path of vertices that represent loops as in Fig 3. Such vertices have additional descendant vertices representing groups of unpaired nucleotides around a loop. Moreover, a vertex in the path representing a bulge has one child vertex with polynomial *x*^*k*^ + *z*, indicating the group of unpaired nucleotides. A vertex in the path representing an interior loop has two child vertices with polynomials *x*^*k*^ + *z* and $x^{k^{\prime }}+z$, representing the two groups of unpaired nucleotides. Here, the exponents *k* and *k*′ indicate the number of unpaired nucleotides in each group. In a type 2 (or type 4) tree-polynomial, an interior loop provides two polynomial factors *x*^*k*^ + *z* and $x^{k^{\prime }}+z$, while a bulge provides only one polynomial factor *x*^*k*^ + *z*, and the coefficient sums increase exponentially with respect to the number of such polynomial factors.

We observe that the linear branches of RNA secondary structures that contribute to the major peaks in type 2 and type 4 coefficient sums have more interior loops than bulges (S11 and S12 Figs). For comparison, the regions where R-loops are rarely formed, at the end of the amplicon region, the RNA secondary structures contain few linear branches, and most of the linear branches have longer stems separating fewer bubbles (S13 Fig). Compared to secondary structures corresponding to the major peaks, the structures corresponding to the minor peaks have fewer bubble-rich linear branches, and more bubbles are bulges rather than interior loops (S14 Fig).

Based on these observations, we postulate that the base pairs in bubble-rich linear branches are prone to breaking. Short stems separated by bubbles are easier to denature as the RNA invades the DNA and starts branch migration. Even short unpaired RNA segments of one or two nucleotides could start the process [22], and short stems make the branch migration easier such that the RNA:DNA duplex can elongate faster. Moreover, we observe that major peaks of R-loop formation contain RNA secondary structures with more interior loops than bulges, and minor peaks contain structures with more bulges than interior loops. Our results suggest that RNA secondary structures (in major peaks) with more interior loops than bulges are easier to break in negatively supercoiled plasmids, and in hyper-negatively supercoiled plasmids. The structures (in minor peaks) with more bulges also form R-loops. This may be a result of base-braking within the DNA duplex which is observed in hyper-negatively supercoiled structures [30]. In type 2 or type 4 scaled sums, the structures with more bulges than interior loops have (exponentially) smaller contribution to the coefficient sums. This partially explains why type 2 and type 4 scaled sums do not capture the minor peaks observed in hyper-negatively supercoiled plasmids. In type 1 scaled sums, bulges and interior loops have equal contribution, and type 1 scaled sums capture structures with more bulges than interior loops. These structures are not associated with R-loops in negatively supercoiled plasmids, and this partially explains why type 1 scaled sums have lower PCCs.

## Discussion

Rooted trees are convenient objects used to represent RNA secondary structures without pseudoknots. In this work, we systematically defined eight different rooted tree representations that record loop-stem relation and combinations of three features (loop size, stem size, loop group) of RNA secondary structures. The eight rooted trees include the previously studied loop-stem (type 1) tree [11] and arc (type 6) tree [12], as well as new tree representations (types 2, 3, 4, 5, 7 and 8). In particular, type 2 and type 4 trees can differentiate RNA secondary structures that are indistinguishable by other studied trees. We introduced tree-polynomial RNA secondary structure representations by applying the tree distinguishing polynomial *P* [17] to type 1, 3, 5, 6, 7 and 8 trees. We generalized polynomial *P* to a newly developed tree distinguishing polynomial *Q*, which better captures the loop group feature of RNA secondary structures with type 2 and 4 trees. These novel representations of RNA secondary structures enable fast and accurate analysis of extensive RNA secondary structures with modern data analytic tools. Moreover, they can be generalized for DNA/RNA nanotechnology and to analyze structures constructed by strand hybridization that depend on branch migration such as RNA origami [31, 32].

In this paper, we first benchmark the performance of tree-polynomial representations in distinguishing different RNA secondary structures by an experiment of clustering non-coding RNA (ncRNA) secondary structures with distance-based clustering methods. The distance methods based on tree-polynomial coefficients accurately clustered ncRNA secondary structures with respect to the families of ncRNAs. We observed that ncRNA secondary structures in the same family but from different organisms have similar loop-stem relations and stem sizes, but may differ in their loop sizes. This resulted in type 1 and type 3 tree-polynomials having the lowest mean misclassification rates and suggests that analyzing different structures requires different tree-polynomials.

Next, we analyzed the link between co-transcriptional RNA secondary structures and R-loop formation. We found strong positive correlation between the probabilities of R-loop formation and the type 1, 2 and 4 scaled coefficient sums computed from corresponding tree-polynomials of the nascent RNA secondary structures. Our results indicate that the scaled coefficient sums associated with the RNA sequence are predictive of R-loop formation.

The tree-polynomials and the coefficient sums helped in identifying RNA secondary structure features that contribute to large coefficient sums. RNA secondary structures with large type 1, 2 and 4 coefficient sums have substructures consisting of many linear branches with short stems separated by multiple bulges and interior loops. We propose that such substructures make the base pairing within the branches energetically less stable, thus aiding the branch migration of the nascent RNA strand interacting with the DNA template strand during transcription, hence increasing the probability of R-loop formation.

We established strong correlations between the probabilities of R-loop formation and the type 1, 2 and 4 scaled sums for plasmids with different topologies: negatively supercoiled or hyper-negatively supercoiled. Type 2 and 4 scaled sums have the strongest correlations with the experimental probabilities for supercoiled plasmids. Type 1 scaled sums provide less strong correlations, but they can indicate the positions of the minor R-loop peaks that are more prevalent for hyper-negatively supercoiled plasmids. Note that our current computational pipeline does not take into account the topology of a plasmid. The topology of a plasmid is known to affect the R-loop formation through additional breaks of the base-pairing [30]. These considerations require new approaches in the studies of DNA topology. At the time of writing and to the best of our knowledge, publicly available data from single molecule footprint experiments that determine R-loop formation up to a nucleotide resolution are only limited to those conducted for pFC8 and pFC53 [6, 28, 29]. This restricted our analysis to these two plasmids.

We used the recent co-transcriptional RNA folding model DrTransformer [21] to obtain the secondary structures of 200nt long segments of the RNA transcript of the amplicon region of each plasmid. In doing this, we assumed that the secondary structures of distinct RNA segments are mutually independent. Ideally, we would focus on co-transcriptional RNA secondary structures of the full amplicon region. However, the amplicon regions of pFC8 and pFC53 are over 1000nt long which would require DrTransformer to process more than 500 RNA sequences with over 600nt. DrTransformer suggests an input sequence shorter than 600nt as it may lose its accuracy. The computation time for a 200nt sequence is approximately 30 minutes on a single 3.2 GHz CPU core. Thus, we choose to restrict our sliding window to 200nt.

Note that while a tree representation of an RNA secondary structure is associated with a unique polynomial *P* (or *Q*), different RNA secondary structures can have the same tree representation. For example, if we swap the 5’ and 3’ ends of an RNA secondary structure, we can have a different secondary structure but identical rooted tree representation. Differentiating such RNA secondary structures requires more detailed tree representations. In future work, we aim to modify the definitions of the polynomials and the rooted tree representations to capture more information about RNA secondary structures, for example, the order of stems and groups of unpaired nucleotides around a multiloop or the opening region. Furthermore, we can also apply the polynomials to other labeled, semi-labeled or unlabeled tree representations of RNA secondary structures. See [19] for an example of a complete polynomial invariant for labeled trees.

The tree-polynomials studied in this paper only work for RNA secondary structures without pseudoknots. In [33], the authors introduced an algebraic language for representing RNA secondary structures with pseudoknots. In order to study secondary structures with pseudoknots using polynomial-based methods, we can represent the secondary structures by directed acyclic graphs (DAGs). Recent studies have introduced complete polynomial invariants for some classes of DAGs in studying phylogenetic networks [34–36]. It may be of interest to apply our pipeline to polynomials associated to DAGs representing RNA structures with pseudoknots.

## Materials and methods

### Background

#### RNA secondary structures

RNA molecules fold into secondary structures that can be described by paired and unpaired nucleotides. The paired nucleotides form double helices interspersed with structures of unpaired nucleotides [10]. We use the terms defined in [20] to describe different regions of an RNA secondary structure. See Fig 1 for an example of an RNA secondary structure and its regions and substructures.

A *stem* refers to an uninterrupted region of base pairs; the two backbones are the *sides* of the stem. The space between two consecutive base pairs and their backbones is a *stem region*. A *hairpin loop* is the region bounded by a single strand of unpaired nucleotides and a stem, where both ends of the string of the unpaired nucleotides form a base pair at the stem. A *bulge* is the region bounded by a single strand of unpaired nucleotides and two stems, where both ends of the string of unpaired nucleotides attach to the same side of the two stems, and the other side of the two stems is an unbroken backbone. An *interior loop* is a region bounded by two strings of unpaired nucleotides and two stems, where two strings of unpaired nucleotides are flanked by the stems. A *multiloop* is the region bounded by *m* strings of unpaired nucleotides and *m* stems, for *m* > 2, such that the strings of unpaired nucleotides and the stems are alternately connected and form a cycle. The *opening region* is the unbounded region containing the 5’ and 3’ ends of the RNA molecule. A *loop* of an RNA secondary structure refers to a hairpin loop, a bulge, an interior loop, a multiloop or to the opening region. If a stem has nucleotides that belong to a loop, then we say that the stem is *connected* to the loop. A stem connects two loops, one close to and the other away from the opening region. A *branch* is a substructure consisting of a stem and the loop away from the opening region, together with all regions connected to the loop. A branch is *linear* if it contains only stems, bulges, interior loops and hairpin loops. A linear branch contains no multiloops.

#### Representations of RNA secondary structures

An RNA secondary structure is often encoded using a *dot-bracket notation* written in parallel with the RNA sequence. A dot represents an unpaired nucleotide, and a pair of brackets represent a pair of nucleotides. In Fig 1, we show the sequence and the dot-bracket notation of the secondary structure of a 41 nt long RNA molecule.

RNA secondary structures are also represented using graphs, especially rooted trees. An intuitive rooted tree representation is the *loop-stem tree*. Every vertex in a loop-stem tree represents a loop of the RNA secondary structure. The root vertex represents the opening region. An edge represents a stem of the RNA secondary structure. Two vertices *v*_1_ and *v*_2_ are connected by an edge *e* in the loop-stem tree if the two loops corresponding to *v*_1_ and *v*_2_ are connected by the stem represented by *e*. This and other graph representations of RNA secondary structures are reviewed in [11].

The dot-bracket notation can be displayed as an arc diagram associated with a unique rooted tree [12], called the *arc tree* of the RNA secondary structure. Leaf vertices of an arc tree represent unpaired nucleotides in the secondary structure. An internal vertex represents either a loop or a stem region. Two internal vertices *v*_1_ and *v*_2_ are connected by an edge *e* in an arc tree if the loops or the stem regions represented by *v*_1_ and *v*_2_ are adjacent and separated by a single base pair. An edge *e* in an arc tree connects a leaf vertex *u* with an internal vertex *v* if the unpaired nucleotide represented by *u* is among the ones that bound the loop represented by *v*.

In Results, we define eight rooted tree representations of RNA secondary structures based on four structural features. These representations include the loop-stem tree and the arc tree.

It is possible for nucleotides in different loops to be connected via hydrogen bonds. The substructure constructed by such non-nested base pairs is called a *pseudoknot* [20]. See Fig 1 for an example. The bonds that define a pseudoknot are not reflected in the standard dot-bracket notation of an RNA secondary structure, the loop-stem tree, nor the arc tree. Other mathematical structures such as directed acyclic graphs are needed to represent pseudoknots. Here, we restrict our study to RNA secondary structures without pseudoknots.

#### A tree distinguishing polynomial

In [17], the author introduced a bivariate graph polynomial that distinguishes trees; we refer to it as *polynomial P*. A unique polynomial *P*(*T*, *x*, *y*) is associated with a given rooted tree *T* through the following process that recursively assigns bivariate polynomials to vertices of *T*, starting from the leaf vertices to the root vertex. Let *v* be a vertex in *T*. If *v* is a leaf vertex, then *P*(*v*, *x*, *y*) = *x*. If *v* is an internal vertex with *k* child vertices *u*_1_, *u*_2_, …, *u*_*k*_, then $P\left(v,x,y\right)=y+\Pi_{i=1}^{k}P\left(u_{i},x,y\right)$. The polynomial for the entire tree *T* is the polynomial of the root vertex *r*, i.e. *P*(*T*, *x*, *y*) = *P*(*r*, *x*, *y*). Fig 3 illustrates the recursive process on type 1 and type 3 tree representations. Polynomial *P* can be directly applied to any rooted tree representation of RNA secondary structures without pseudoknots. In [17], the author proved that polynomial *P* is a complete invariant for trees, i.e. two trees *T*_1_ and *T*_2_ are isomorphic if and only if *P*(*T*_1_, *x*, *y*) = *P*(*T*_2_, *x*, *y*). Hence, polynomial *P* distinguishes the RNA secondary structures represented by the trees. Polynomial *P* is interpretable [17]. More precisely, every term in *P*(*T*, *x*, *y*) corresponds to a subtree of *T*, thus every term in *P*(*T*, *x*, *y*) describes a substructure of the RNA secondary structure represented by *T*.

#### Polynomial distance between trees

To compare and analyze RNA secondary structures represented by trees, we use a polynomial distance based on the Canberra distance as in [18]. A polynomial *P*(*T*, *x*, *y*) of degree *n* can be represented as an (*n* + 1) × (*n* + 1) coefficient matrix, where the entry *c*^(*i*,*j*)^ at the *j*-th row and the *i*-th column is the coefficient of the term *c*^(*i*,*j*)^*x*^*i*^*y*^*j*^ in *P*(*T*, *x*, *y*), for 0 ≤ *i*, *j* ≤ *n*. Let *S* and *T* be two rooted trees and *b*^(*i*,*j*)^ and *c*^(*i*,*j*)^ be the coefficients of the corresponding terms in *P*(*S*, *x*, *y*) and *P*(*T*, *x*, *y*), respectively. The polynomial distance between *S* and *T* is defined by Formula (3), where the function *κ* is defined by Formula (2) in Results.

$$\begin{array}{c} d_{P}\left(S,T\right)=\sum_{0\leq i,j\leq n}\kappa \left(b^{\left(i,j\right)},c^{\left(i,j\right)}\right) \end{array}$$ (3)

We call this distance the *polynomial P distance*. In Results, we generalize polynomial *P* and polynomial *P* distance to represent and compare newly introduced tree representations of RNA secondary structures.

### Data and experiments

#### Non-coding RNA secondary structures

We acquired data of RNA secondary structures from the bpRNA-1m database, which is constructed with the tool bpRNA that parses RNA structures from seven different sources [20]. We use one of the sources, Rfam, to construct a dataset of RNA secondary structures for our study. Rfam is a database that contains more than a thousand families of non-coding RNAs (ncRNAs) [24]. We construct a dataset by selecting 735 secondary structures without pseudoknots from seven families of ncRNAs chosen to have sequences of similar length with distinct secondary structures. We use this dataset to benchmark the performance of the tree-polynomial distance for distinguishing RNA secondary structures and refer to this dataset as the *bpRNA-Rfam-7* dataset. See S1 and S2 Tables.

Given a choice of tree-polynomial representation, we first cluster the 735 ncRNA secondary structures in the bpRNA-Rfam-7 dataset with tree-polynomial distances, then we compare the clustering results with the true families of the ncRNA secondary structures. Specifically, we compute tree-polynomial distances between each pair of secondary structures in the bpRNA-Rfam-7 dataset for a given tree-polynomial representation. We apply the k-medoids clustering algorithm [27] to the pairwise tree-polynomial distances. The k-medoids clustering algorithm is unsupervised, i.e. it assigns an integer between 1 and 7 to each ncRNA secondary structure. We use misclassification rates to compare the classes of RNA secondary structures determined by the clustering algorithm to the true families of the ncRNAs. The misclassification rate for an unsupervised clustering experiment can be computed by the majority rule [18]. Specifically, we associate a true family of ncRNA secondary structures with a cluster *i* if the majority of ncRNA secondary structures in cluster *i* are from the true family, where *i* is an integer between 1 and 7. Any secondary structure in cluster *i* that is not from the associated ncRNA family is misclassified. The misclassification rate of this clustering experiment is the percentage of misclassified secondary structures among the 735 ncRNA secondary structures in the bpRNA-Rfam-7 dataset. Since the k-medoids algorithm is heuristic, we repeat the clustering experiment 1000 times for each tree-polynomial representation and compute the mean misclassification rate (MMR) over the 1000 experiments.

Additionally, we extend our clustering experiment to a larger dataset constructed by selecting all Rfam RNA secondary structures of length between 100nt and 200nt without pseudoknots available in the bpRNA-1m database. There are currently 16,039 such RNA secondary structures constructed using comparative sequence analysis of bpRNA [20]. We refer to this dataset as the *bpRNA-Rfam-large* dataset.

#### Plasmids containing R-loop forming genes

We use sequence data of DNA plasmids containing genes that are known to form R-loops: pFC8 of length 3669bp and pFC53 of length 3906bp. These plasmids were studied in [6]. In our analysis, we only use the amplicon regions between nucleotides 81–1512 in pFC8 and 81–1829 in pFC53. Here, the *sequence of a plasmid* refers to the sequence of the amplicon region. We also use experimental data of the two circular plasmids with different topologies: negatively supercoiled and hyper-negatively supercoiled. These data were obtained by SMRF-seq based R-loop footprinting [28, 29] and reported in [6, 37]. In the experiments, the circular plasmids that were negatively or hyper-negatively supercoiled were linearized after transcription and before SMRF-seq footprinting. Given a plasmid and its topology, the probability of R-loop formation at each nucleotide can be obtained from the experimental data.

#### Sequence processing and structure obtaining for R-loop plasmids

First, we convert the DNA sequence of a plasmid to its corresponding RNA sequence after transcription, which we refer to as the *RNA sequence of the plasmid*. Given an RNA sequence of a plasmid with *n* nucleotides, we take *m*-nucleotide long segments (*m* < *n*) starting at the 5′ end and consider RNA secondary structures for each *m*-size segment at every nucleotide *i*. Specifically, for every integer *i*, 1 ≥ *i* ≤ *n* − *m* + 1, we consider the RNA segment in interval [*i*, *i* + *m* − 1]. For example, for pFC8, we have *n* = 1512 − 81 + 1 = 1432, and if we set *m* = 200, then the RNA segments considered are in intervals [1, 200], [2, 201], …, [1233, 1432]. For each segment thus obtained, we use DrTransformer to obtain the co-transcriptional RNA secondary structures [21]. For an *m*-long segment, DrTransformer generates a series of minimum energy secondary structures for each of the segment interval [1, *j*], where *j* = 1, 2, …, *m*. Thus, every segment has a series of *m* secondary structures with 1 to *m* nucleotides. This series of structures reflect RNA structural changes during transcription. For an RNA sequence of *n* nucleotides and *m*-long segments, DrTransformer produces *m*(*n* − *m* + 1) secondary structures. We index these structures with two-dimensional coordinates (*i*, *j*), where *i* indicates the segment [*i*, *i* + *m* − 1] of the RNA sequence, and *j* indicates the *j*th transcription step of the sequence producing the structure for [*i*, *i* + *j* − 1]. Since DrTransformer requires large computing power for each segment, in this work we bound *m* = 200.

#### Computing tree-polynomial coefficient-sums for R-loop plasmids

Given a plasmid’s RNA sequence with *n* nucleotides, we compute the type *k* for *k* = 1, …, 8 tree-polynomial representation for each of the *m*(*n* − *m* + 1) RNA secondary structures, where *m* is the segment length. For each structure indexed (*i*, *j*), we compute the coefficient sum of the type *k* tree-polynomial representation, and denote this coefficient-sum with *s*(*i*, *j*). To compare with the R-loop formation probabilities of a plasmid, we introduce the following variations of coefficient-sums of secondary structures. The *normalized coefficient sum*, or simply the *normalized sum* of the secondary structure at (*i*, *j*) is defined to be *s*(*i*, *j*)/max_1≤*ℓ*≤*n*−*m*+1_ *s*(*ℓ*, *j*). Recall that the R-loop formation probability of a plasmid is assumed to be a function of its sequence. In order to simulate the process in SMRF-seq of constructing the probability of R-loop formation [6, 28, 29], we define the *overlapping sum* for a given plasmid at the transcription step *j*. For each *j*, the overlapping sum is a function assigning a real number to each nucleotide in the RNA sequence.

For every integer *i* in the interval [1, *n* − *m* + 1] we assign *s*(*i*, *j*) to every nucleotide between the *i*-th and the (*i* + *m* − 1)-th nucleotide (including the end nucleotides). Then, we add all coefficient sums assigned at every nucleotide of the RNA sequence. The result is the overlapping sum of the plasmid at transcription step *j*. See S7 Fig for an illustration of the process. By dividing the overlapping sum of the RNA sequence at transcription step *j* by the maximum value of the overlapping sum, we have the *normalized overlapping sum* of the RNA sequence at transcription step *j*. For a better comparison with the experimental results, we multiply the normalized overlapping sum with the maximum value of the experimentally obtained probability for R-loop formation to obtain the *scaled overlapping sum*, or simply the *scaled sum* of the RNA sequence of the plasmid. For a plasmid, we compare the scaled sum of the RNA sequence at transcription step *j* with the experimentally obtained R-loop formation probability.

In summary, first we construct 200nt sequence segments of the nascent RNA strand from the DNA sequences of the two plasmids, pFC8 and pFC53, and use DrTransformer to obtain co-transcriptional RNA secondary structures for the RNA segments. Next, for *k* = 1, …, 8 we compute the type *k* tree-polynomial representations of the RNA secondary structures and associate the respective type *k* coefficient sum. For a transcription step *j*, we compute the type *k* overlapping sum, especially the type *k* scaled overlapping sum, in order to compare with the experimentally obtained probabilities of R-loop formation. In our computation we focus on transcription steps *j* = 191, …, 200. The experimental data contains R-loops obtained from plasmids with two different DNA topologies: supercoiled and hyper-negatively supercoiled. We compare the type *k* scaled sums with the probabilities of R-loop formation for both topologies. See S8 Fig for a diagram displaying the entire computational pipeline.

## Supporting information

S1 Appendix Supplementary material. This file includes additional information about the definitions, data, experiments and results of this paper. It contains details about the following supporting figures and tables. This file also includes additional Fig A showing the computational time of each tree-polynomial representation, Fig B displaying an example of misclassified ncRNA secondary structure, Fig C showing that the scaled sums are not correlated with DrTransformer’s minimum free energy outputs, and Figs D, E, and F which show results of the type 3 tree-polynomial representation in analyzing R-loop formation.
(PDF)

S1 Fig Rooted tree and polynomial representations of RNA secondary structures. The figure shows the last four rooted tree representations and their corresponding polynomial representations of the RNA secondary structure displayed in Fig 1. Vertices in the trees are colored based on the loops or stem regions that they represent, and the black round vertices represent unpaired nucleotides. The leaf vertices in type 7 and type 8 tree representations represent artificial vertices introduced for grouping unpaired nucleotides. Alongside every tree representation, the recursive process of computing the corresponding polynomial from the leaf vertices to the root vertex is displayed. The polynomial at the root vertex of a rooted tree is the polynomial that represents the tree.
(TIF)

S2 Fig An example of a pair of RNA secondary structures that requires type 2 and type 4 tree representations to distinguish. Panel A and panel B show a pair of 52nt long RNA secondary structures that are the same in loop-stem relation, loop size and stem size but different in loop group. Each panel also displays the corresponding type 1, type 5 and type 2 tree representations, where only type 2 tree representations distinguish between the RNA secondary structures.
(TIF)

S3 Fig Visualization of pairwise tree-polynomial distances between ncRNA secondary structures in the bpRNA-Rfam-7 dataset. The top two panels show the MDS plots of the pairwise polynomial *P* distances between type 1 (panel A) and type 2 (panel B) tree-polynomial representations of the 735 ncRNA secondary structures in the bpRNA-Rfam-7 dataset. The bottom panels show the analogous MDS plots of the pairwise polynomial *Q* distances between type 3 (panel C) and type 4 (panel D) tree-polynomial representations. Each dot in a panel represents an ncRNA secondary structure of the ncRNA family corresponding to its color. We observe distinct clusters for each of the seven families of ncRNAs in all four plots. The plot for type 3 tree-polynomial representations corresponds to the best clustering results, with only two U3 small nucleolar RNAs (cyan) close to other clusters.
(TIF)

S4 Fig Visualization of pairwise tree-polynomial distances between ncRNA secondary structures in the bpRNA-Rfam-7 dataset. The figure shows the MDS plots of the pairwise polynomial *P* distances between type 5 (panel A) and type 6 (panel B) tree-polynomial representations of the 735 ncRNA secondary structures in the bpRNA-Rfam-7 dataset, and the MDS plots of the pairwise polynomial *P* distances between type 7 (panel C) and type 8 (panel D) tree-polynomial representations of the ncRNA secondary structures in the bpRNA-Rfam-7 dataset. Each dot in a panel represents an ncRNA secondary structure of the ncRNA family corresponding to its color.
(TIF)

S5 Fig Examples of secondary structures from the same ncRNA family in the bpRNA-Rfam-7 dataset. This figure shows examples of 5.8S ribosomal RNA secondary structures in the bpRNA-Rfam-7 dataset. We obtained the secondary structure diagrams and information from bpRNA-1m.
(TIF)

S6 Fig Examples of secondary structures from the same ncRNA family in the bpRNA-Rfam-7 dataset. This figure shows examples of U12 minor spliceosomal RNA secondary structures in the bpRNA-Rfam-7 dataset. We obtained the secondary structure diagrams and information from bpRNA-1m.
(TIF)

S7 Fig Visualization of the process for computing normalized and scaled overlapping sums. Panel A shows the probability of R-loop formation (blue) as a function of the RNA sequence, segments of the RNA sequence and the lists of RNA secondary structures (red and purple) of the first and the last RNA segments. Panel B shows the coefficient sums (black numbers) and the normalized sums (gray dots and horizontal lines) of the RNA segments at a transcription step and the process of computing the overlapping sum (yellow numbers below the sequence), normalized overlapping sum (yellow curve) and the scaled sum (black curve) of the RNA sequence from the coefficient sums.
(TIF)

S8 Fig A diagram of the computational pipeline for analyzing the link between RNA secondary structures and R-loop formation. This diagram shows how we process the DNA sequence data of the two plasmids and compare the obtained RNA secondary structure with the probabilities of R-loop formation.
(TIF)

S9 Fig The correlations between the scaled sums and the R-loop formation probabilities of hyper-negatively supercoiled pFC8 and pFC53 plasmids. The figure shows the experimental probability of R-loop formation for the hyper-negatively supercoiled pFC8 plasmid with the type 1 (panel A) and the type 4 (panel B) scaled sums, and the experimental probability of R-loop formation for the hyper-negatively supercoiled pFC53 plasmid with the type 1 (panel C) and the type 4 (panel D) scaled sums. The experimental probabilities of R-loop formation are from [6]. The displayed scaled sums have the highest PCC in the last 10 transcription steps.
(TIF)

S10 Fig The correlations between the type 2 scaled sums and the R-loop formation probability of pFC8 and pFC53 plasmids. The figure shows the correlations between the type 2 scaled sums (with the highest PCC in the last 10 transcription steps) and the R-loop formation probabilities of the supercoiled pFC8 plasmid (panel A) and of the hyper-negatively supercoiled pFC8 plasmid (panel B), and the correlations between the type 2 scaled sums (with the highest PCC in the last 10 transcription steps) and the R-loop formation probabilities of the supercoiled pFC53 plasmid (panel C) and of the hyper-negatively supercoiled pFC53 plasmid (panel D).
(TIF)

S11 Fig Secondary structures of RNA segments that have the largest type 2 coefficient sums. The figure shows the secondary structures of RNA segments of the pFC8 plasmid (panel A and B) and the pFC53 plasmid (panel C and D) with the two largest type 2 coefficient sums at the transcription step with the highest PCC in the last 10 transcription steps. Type 2 tree representations are displayed following the corresponding secondary structures.
(TIF)

S12 Fig Secondary structures of RNA segments that have the largest type 4 coefficient sums. The figure shows the secondary structures of RNA segments of the pFC8 plasmid (panel A and B) and the pFC53 plasmid (panel C and D) with the two largest type 4 coefficient sums at the transcription step with the highest PCC in the last 10 transcription steps. Type 4 tree representations are displayed following the corresponding secondary structures.
(TIF)

S13 Fig Secondary structures of RNA segments near the 3’ end of the amplicon region. The figure shows the secondary structures of RNA segments of the pFC8 plasmid (panel A and B) and the pFC53 plasmid (panel C and D) near the 3’ end of the amplicon region that have the two largest type 1 coefficient sums at the transcription step with the highest PCC in the last 10 transcription steps. Type 1 tree representations are displayed following the corresponding secondary structures.
(TIF)

S14 Fig Secondary structures of RNA segments in the minor peak of R-loop formation. The figure shows the secondary structures of RNA segments of the pFC8 plasmid (panel A and B) and the pFC53 plasmid (panel C and D) in the minor peak of R-loop formation that have the two largest type 1 coefficient sums at the transcription step with the highest PCC in the last 10 transcription steps. Type 1 tree representations are displayed following the corresponding secondary structures.
(TIF)

S1 Table The bpRNA-Rfam-7 dataset of non-coding RNA (ncRNA) secondary structures. The first column shows the seven Rfam ncRNA families selected in the study. The second column shows the number of RNA secondary structures without pseudoknots in each family. The third column shows the average length of the ncRNA sequences in each family.
(PDF)

S2 Table The bpRNA IDs of ncRNA secondary structures in the bpRNA-Rfam-7 dataset. The table shows the bpRNA ID of the secondary structures from the seven ncRNA families in the Rfam dataset. The asterisk superscript indicates that there are five U3 small nucleolar RNA secondary structures (with ID 2946, 2951, 2965, 2970 and 2998) that have pseudoknots and are not included in the bpRNA-Rfam-7 dataset.
(PDF)

S3 Table Mean misclassification rates of clustering RNA secondary structures in the bpRNA-Rfam-large dataset. This table shows the mean misclassification rates (MMRs) of clustering all 16,039 Rfam RNA secondary structures with length between 100nt and 200nt and without pseudoknots in the bpRNA-1m database.
(PDF)

S4 Table The highest Pearson’s correlation coefficients between the scaled sums and the probabilities of R-loop formation of the last 10 transcription steps. The first columns show the types of tree-polynomial representations. The last four columns show the highest Pearson’s correlation coefficients (PCCs) between the scaled sums of the tree-polynomial representations and the probabilities of R-loop formation of the corresponding plasmids in the last 10 transcription steps. Inside the parentheses after the PCCs are the transcription steps of the corresponding scaled sums.
(PDF)

S5 Table The highest Pearson’s correlation coefficients between the scaled sums and the probabilities of R-loop formation in all transcription steps. The first columns show the types of tree-polynomial representations. The last four columns show the highest Pearson’s correlation coefficients (PCCs) between the scaled sums of the tree-polynomial representations and the probabilities of R-loop formation of the corresponding plasmids over all transcription steps. Inside the parentheses after the PCCs are the transcription steps of the corresponding scaled sums.
(PDF)
