# Supplementary material for: Tree polynomials identify a link between co-transcriptional R-loops and nascent RNA folding
Source: PLoS Comput Biol. 2024 Dec 13;20(12):e1012669. doi: 10.1371/journal.pcbi.1012669 (PMC11706388; doi:10.1371/journal.pcbi.1012669)
Supplement: S1 Appendix — This file includes additional information about the definitions, data, experiments and results of this paper. It contains details about the following supporting figures and tables. This file also includes additional Fig A showing the computational time of each tree-polynomial representation, Fig B displaying an example of misclassified ncRNA secondary structure, Fig C showing that the scaled sums are not correlated with DrTransformer’s minimum free energy outputs, and Figs D, E, and F which show results of the type 3 tree-polynomial representation in analyzing R-loop formation. (PDF) [file pcbi.1012669.s001.pdf]

Supplementary material of “Tree polynomials identify a link between co-transcriptional R-loops and nascent RNA folding”

Pengyu Liu[1], Jacob Lusk[1], Nataša Jonoska[3], Mariel Vázquez[1,2*]

1 Department of Microbiology and Molecular Genetics, **2** Department of Mathematics, University of California, Davis, Davis, California, United States of America
3 Department of Mathematics and Statistics, University of South Florida, Tampa, Florida, United States of America

* mrlvazquez@ucdavis.edu

Tree-polynomial representations

We study four important features of RNA secondary structures: loop-stem relation, loop size, stem size and loop group. All rooted tree representations record the loop-stem relation. There are eight possible ways to record combinations of the three other features. Type 7 tree representations record loop-stem relation and loop group, and type 8 tree representations record loop-stem relation, stem size and loop group. The first four tree representations are displayed in Fig 3 and the last four tree representations are displayed in S1 Fig.

Here, we describe how we construct the eight tree representations from the RNA secondary structures. The loops in the RNA secondary structure are represented by the colored vertices in the tree representations in Fig 3 and S1 Fig. Vertex color corresponds to the type of loops; see Fig 1. The tree representations that record loop size (type 2, type 4, type 5 and type 6) have black leaf vertices that represent unpaired nucleotides around loops. Let L be a loop and $n_1, n_2, \ldots, n_k$ be the unpaired nucleotide around the loop L. The black leaf vertices that represent $n_1, n_2, \ldots, n_k$ are descendants of the colored vertex that represents the loop L. The tree representations that record stem size (type 3, type 4, type 6 and type 8) have gray internal vertices that represent stem regions. We represent a stem with n base pairs in the RNA secondary structure by a path only consisting of $n-1$ gray vertices in the tree representations. Since stems do not have unpaired nucleotides, the gray vertices in the tree representations do not have child vertices that are leaf vertices in the trees. The tree representations that record both loop size and loop group (type 2, and type 4) have black square artificial vertices representing the groups of unpaired nucleotides around each loop. Suppose that g is a group of the unpaired nucleotides $n_1, n_2, \ldots, n_k$ around a loop L. The black square artificial vertex that represents group g is a child vertex of the colored vertex representing L. The black leaf vertices that represent unpaired nucleotides $n_1, n_2, \ldots, n_k$ are child vertices of the black square artificial vertex that represents the group g. In S2 Fig, we show an example of an RNA secondary structure that can be distinguished by the type 2 and the type 4 tree representations but not the other six tree representations. The two distinct 52nt long RNA secondary structures have the same loop-stem relation, the corresponding stems have the same size and the corresponding loops have the same number of unpaired nucleotides. The only difference between the secondary structures is how the unpaired nucleotides are grouped around each loop.

The tree representations that record loop group but not loop size (type 7 and type 8) have the artificial vertices that represent groups of unpaired nucleotides as leaf vertices,

so we represent them with the black round vertices; see S1 Fig. Type 7 and the type 8 tree representations provide little additional information about the RNA secondary structure compared to the type 1 and type 3 tree representations respectively. This is because the groups of unpaired nucleotides are divided by branches around a loop. Type 7 and type 8 tree representations have the following properties: a hairpin loop must only have one artificial vertex as a child and leaf vertex; a bulge must have two child vertices including one and only one artificial vertex as a leaf vertex; an interior loop must have three child vertices including two artificial vertices as leaf vertices; a multiloop with n branches must have $2n-1$ child vertices with n artificial vertices as leaf vertices. The only additional information that a type 7 or a type 8 tree representation can provide when compared to a type 1 or a type 3 tree representation is whether the nucleotides at the 5' and the 3' ends are paired or unpaired. Suppose that the opening region of an RNA secondary structure has n branches. If both ends are paired, then the root vertex has $n-1$ artificial vertices as child and leaf vertices. If one end is paired, then the root vertex has n artificial vertices as child and leaf vertices. If both ends are unpaired, then the root vertex has $n+1$ artificial vertices as child and leaf vertices. Moreover, the type 7 and type 8 tree-polynomial representations of an RNA secondary structure have the same coefficient sums as the type 5 and type 6 tree-polynomial representations, respectively. This is because leaf vertices contribute only to the exponents and not to the coefficients. It is for these reasons that we do not discuss type 7 and type 8 tree-polynomial representations in the main text. Type 7 and type 8 tree-polynomial representations also perform the worst in clustering the non-coding RNA secondary structures in the bpRNA-Rfam-7 dataset, with mean misclassification rates 24.32% and 22.16% respectively.

We use polynomial Q to define type 2 and type 4 tree-polynomial representations. Other types of tree representations use the previously studied tree distinguishing polynomial P. The recursive processes of computing the polynomials from the tree representations are illustrated in Fig 3 and S1 Fig. Given a rooted tree T, note that if we take $z = y$ in a polynomial $Q(T, x, y, z)$, then we obtain the polynomial $P(T, x, y)$. Since the polynomial P distinguishes trees, the polynomial Q also distinguishes trees. Mathematically, two trees are isomorphic if and only if they have the same polynomial Q.

In Fig A, we show the average computational time needed to compute each tree-polynomial representation for a secondary structure in the bpRNA-Rfam-7 dataset. The average length of an ncRNA secondary structure in the dataset is 171.97nt; see S1 Table. As expected, computing the 3-variable polynomial Q is more expensive than computing the bivariate polynomial P.

Clustering RNA secondary structures

Basic information of the seven families of non-coding RNA (ncRNA) secondary structures in the bpRNA-Rfam-7 dataset is listed in S1 Table. More detailed information about an individual ncRNA secondary structure in the dataset can be found at the bpRNA-1m database (`https://bprna.cgrb.oregonstate.edu`), where each RNA secondary structure is associated with a bpRNA ID. S2 Table lists the bpRNA IDs of the ncRNA secondary structures in the bpRNA-Rfam-7 dataset.

In S3 Fig and S4 Fig, we visualize pairwise polynomial distances between the tree-polynomial representations of the ncRNA secondary structures with multidimensional scaling (MDS).

In S5 Fig and S6 Fig, we display examples of ncRNA secondary structures from the 5.8S ribosomal RNA family and the U12 minor spliceosomal RNA family in the bpRNA-Rfam-7 dataset. Examples of other secondary structures in the bpRNA-Rfam-7

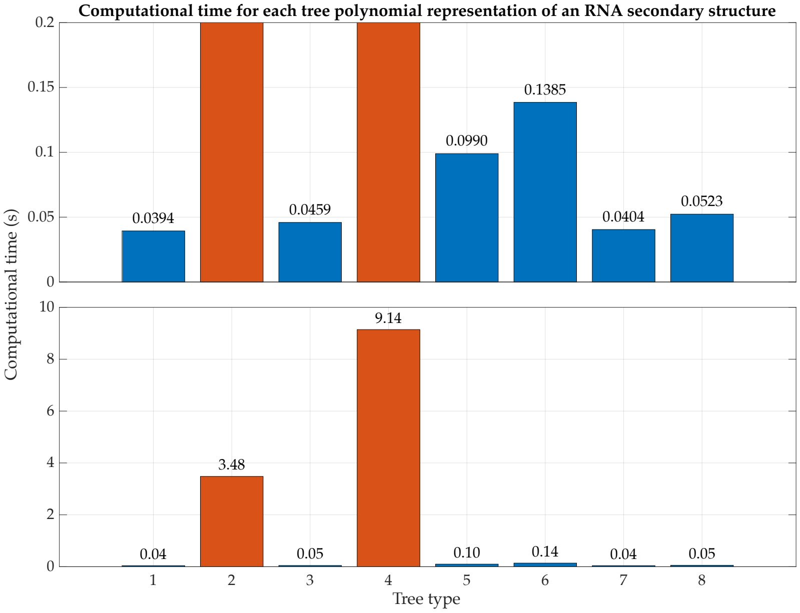

Fig A. Average computational time of each tree-polynomial representation for an RNA secondary structure. Each bar shows the average time spent computing the corresponding tree-polynomial representations of the 735 ncRNA secondary structures (with average length 171.97 nt) in the bpRNA-Rfam-7 dataset on a single 3.2 GHz CPU core. Blue bars indicate that the representations use polynomial P, and red bars indicate that the representations use polynomial Q. The top panel shows a more detailed comparison of the blue bars.

dataset can be found at the bpRNA-1m database with their bpRNA ID. The We observe that ncRNA secondary structures in the same family from different organisms can display large differences in loop size, while their loop-stem relation and stem size are consistent. This partially explains why type 3 and type 1 tree representations perform the best in clustering the ncRNA secondary structures of the bpRNA-Rfam-7 dataset. In Fig B, we show an example of ncRNA secondary structures that are misclassified by the k-medoids algorithm with type 1 and type 3 tree-polynomial distances. The U2 spliceosomal RNA secondary structure (panel A) was misclassified into the group of 5.8 ribosomal RNAs (panel D and E) instead of the group of U2 spliceosomal RNAs (panel B and C).

Additionally, we have performed the k-medoids clustering analysis on the bpRNA-Rfam-large dataset. The results are consistent with analysis on the smaller bpRNA-Rfam-7 dataset and are reported in S3 Table.

RNA secondary structures and R-loop formation

In Materials and methods, we describe the process of computing the overlapping sum, the normalized overlapping sum and the scaled overlapping sum based on the coefficient sums of type k of tree-polynomial representations of RNA segments for an integer k between 1 and 8. We illustrate this process in S7 Fig with a toy example. In this example, the RNA sequence has $n = 14$nt, and the length of a segment is $m = 5$nt, for a total of 10 segments in increments of 1nt; see panel A. For each segment, DrTransformer predicts $m = 5$ co-transcriptional RNA secondary structures from 1nt to 5nt. The

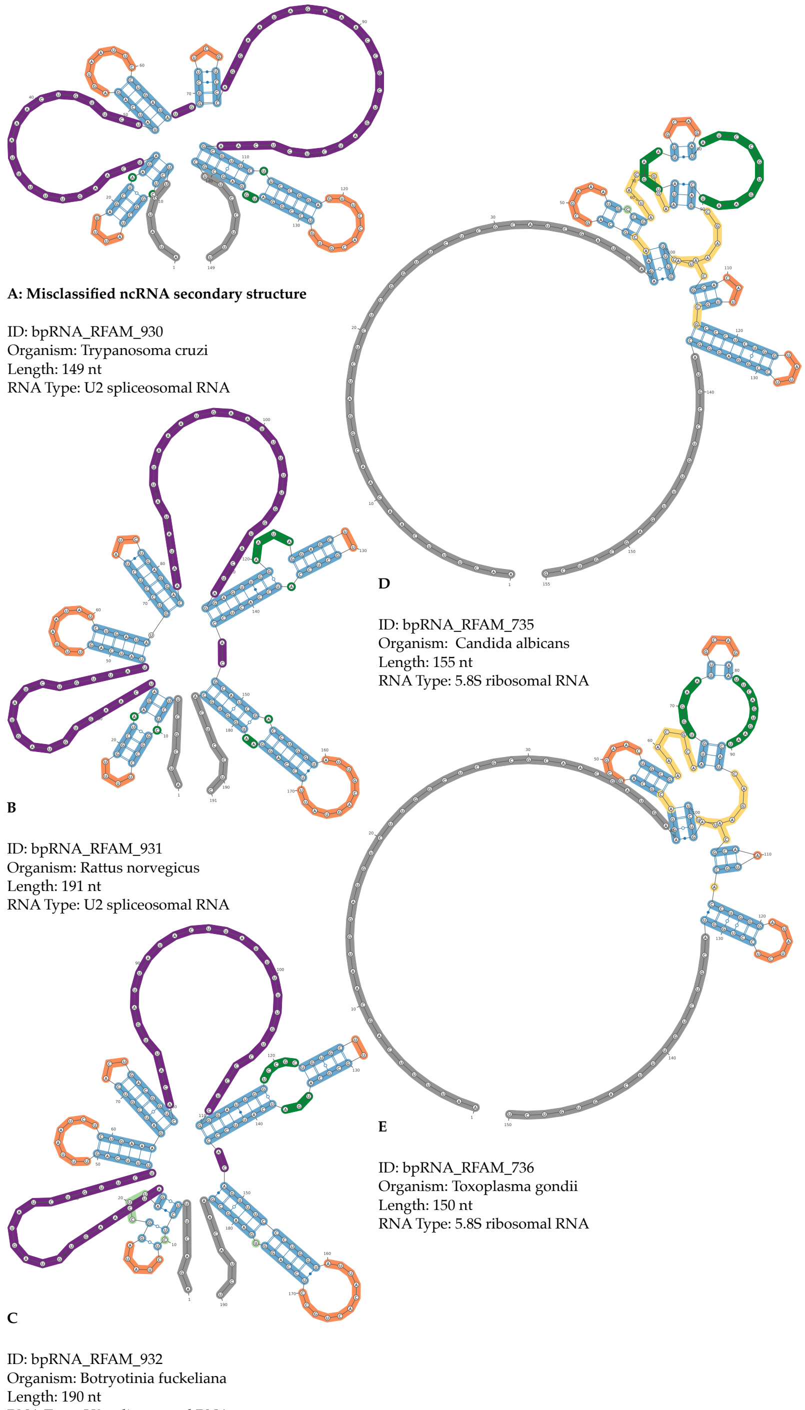

Fig B. An example of misclassified ncRNA secondary structures. This figure shows an ncRNA secondary structure that is misclassified by the k-medoids algorithm with type 1 and type 3 tree-polynomial distances. The U2 spliceosomal RNA secondary structure in panel A was misclassified into the group of 5.8 ribosomal RNAs (panel D and E) instead of the group of U2 spliceosomal RNAs (panel B and C). We obtained the secondary structure diagrams and information from bpRNA-1m.

predicted co-transcriptional RNA secondary structures for the first and the last segment are displayed in panel A. The parameters used in DrTransformer are all at default values in this paper. We take the secondary structures for all segments at transcription step $j = 5$. For the secondary structure of the i-th segment, we compute its tree-polynomial representation and the coefficient sum $s(i, j)$. We put the number $s(i, j)$ at every nucleotide in the i-th segment (panel B). The overlapping sum of the RNA sequence is computed by vertically adding all numbers at each nucleotide (panel B). We divide the overlapping sum by its maximum value (29 in the example) to obtain the normalized overlapping sum of the RNA sequence (the yellow curve in panel B). Then, we multiply the normalized overlapping sum with the highest probability of R-loop formation to obtain the scaled overlapping sum of the RNA sequence (the black curve in panel B).

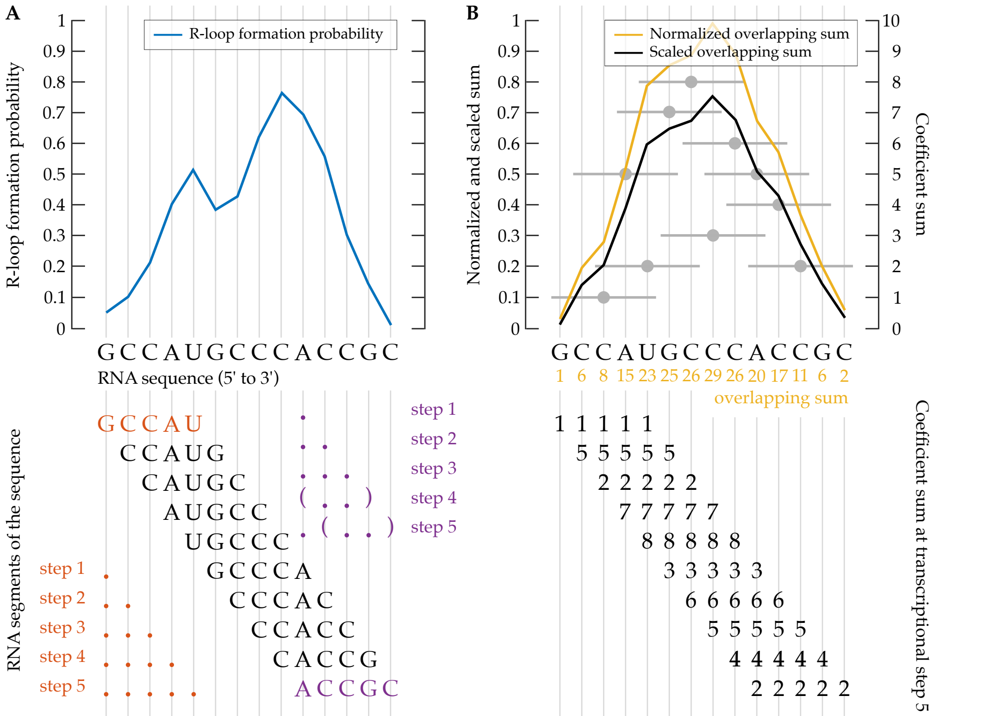

S7 Fig. Visualization of the process for computing normalized and scaled overlapping sums. Panel A shows the probability of R-loop formation (blue) as a function of the RNA sequence, segments of the RNA sequence and the lists of RNA secondary structures (red and purple) of the first and the last RNA segments. Panel B shows the coefficient sums (black numbers) and the normalized sums (gray dots and horizontal lines) of the RNA segments at a transcription step and the process of computing the overlapping sum (yellow numbers below the sequence), normalized overlapping sum (yellow curve) and the scaled sum (black curve) of the RNA sequence from the coefficient sums.

In Materials and methods, we describe pipeline of the computational experiment for analyzing the link between the secondary structures of the nascent RNA strand and the probabilities of R-loop formation. In S8 Fig, we visualize the computational pipeline of how we use the data of the two plasmids to conduct the experiment.

The co-transcriptional RNA folding model, DrTransformer, outputs minimum free energy (MFE) for every predicted nascent RNA structure, and the MFE is a number statistics of the structure's stability. In Fig C, we show the MFE for every RNA

segment and that the scaled sums of tree-polynomials provide different number statistics regarding the nascent RNA secondary structures.

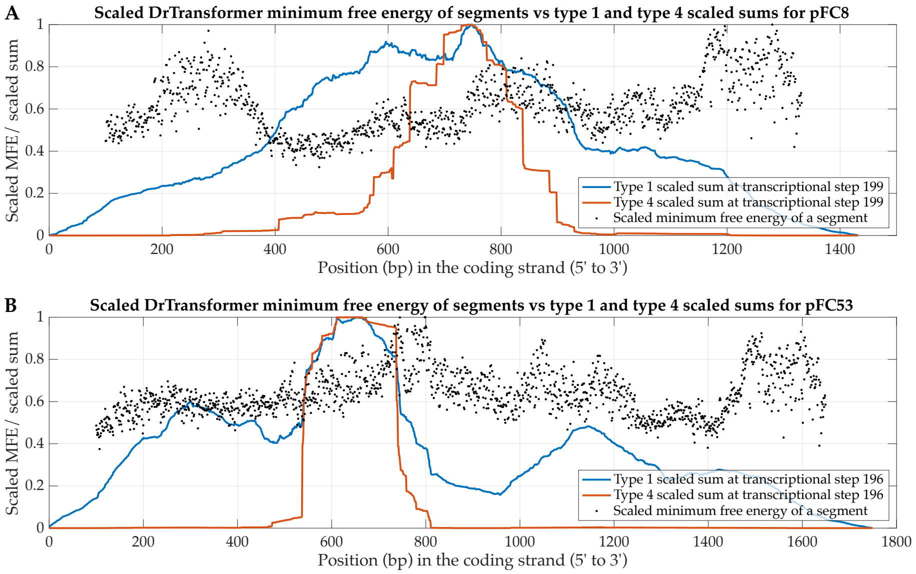

Fig C. Comparison between DrTransformer minimum free energy of segments and type 1 and type 4 scaled sums of pFC8 and pFC53. This figure shows that the scaled sums of tree-polynomials provide different statistics of DrTransformer predicted RNA secondary structures than the minimum free energy. Each black dot indicates the normalized minimum free energy of the DrTransformer predicted secondary structure of the RNA segment centered at the black dot. The minimum free energy is normalized by dividing the maximum value over all RNA segments. Panel A shows the comparison for pFC8 and panel B shows the comparison for pFC53. The Pearson's correlation coefficients between the DrTransformer minimum free energy and the scaled sums are -0.27 (pFC8, type 1), -0.09 (pFC8, type 4), -0.08 (pFC53, type 1) and -0.04 (pFC53, type 4)

In S9 Fig, we replace the probabilities of R-loop formation of supercoiled plasmids in Fig 4 with the probabilities of R-loop formation of hyper-negatively supercoiled plasmids, and show the correlations with the type 1 and type 4 scaled sums. The R-loop formation probabilities of the hyper-negatively supercoiled plasmids have two outstanding peaks, while the R-loop formation probabilities of the supercoiled plasmids have only one outstanding peaks. The higher peaks indicate the position of the major R-loop forming sequence cluster and the lower peaks indicate the position of the minor R-loop forming sequence cluster of each plasmids.

In S10 Fig, we show the correlations between probabilities of R-loop formation of supercoiled and hyper-negatively supercoiled plasmids and the type 2 scaled sums. The type 2 scaled sums are similar to the type 4 scaled sums for the two plasmids. The difference between the two tree representations is that type 4 tree representations record stem size while the type 2 ones do not. In S11 Fig, we show the secondary structures of RNA segments that have the two largest type 2 coefficient sums and the corresponding type 2 tree representations. In S12 Fig, we show the secondary structures of RNA segments that have the two largest type 4 coefficient sums and the corresponding type 4 tree representations.

In Fig D, we show the correlations between R-loop formation probabilities of supercoiled and hyper-negatively supercoiled plasmids and the type 3 scaled sums. Note

that the type 3 tree-polynomial representation records the stem size but not the loop size or loop group with the bivariate polynomial P. The type 3 scaled sums are not strongly correlated with the probabilities of R-loop formation. The type 3 tree representation of a linear branch is a path with vertices representing bubbles and stem regions, which are not differentiated by the tree-polynomial representations. This mixing of information is partially why the type 3 scaled sums and the R-loop formation probabilities are not strongly correlated. The RNA secondary structures with the two largest type 3 coefficient sums are displayed in Figs E and F, where both the type 1 and the type 3 tree representations are shown. We observe that most of the linear branches in these secondary structures do not have many bubbles, and the large type 3 coefficient sums are contributed by the vertices that represent stem regions.

In S13 Fig, we show the secondary structures of RNA segments that have the two largest type 1 coefficient sums near the 3' end of the amplicon region, where few R-loops form. The corresponding type 1 tree representations are displayed following the secondary structures. In S14 Fig, we show the secondary structures of RNA segments that have the two largest type 1 coefficient sums in the minor R-loop forming cluster and the corresponding type 1 tree representations are displayed following the secondary structures.

The results discussed so far only cover the scaled sums that have the highest Pearson's correlation coefficients (PCCs) with the probabilities of R-loop formation in the last 10 transcription steps. Analogous data of the PCCs for type 5 to type 8 tree-polynomial representations are listed in S4 Table. As previously discussed, the type 7 and the type 8 tree-polynomial representations of an RNA secondary structure have the same coefficient sums as the type 5 and type 6 tree-polynomial representations of the secondary structure respectively. Hence, the type 7 and the type 8 scaled sums of the plasmids are identical to the type 5 and the type 6 scaled sums of the plasmids, and they have the same PCCs. In S5 Table, we list the PCCs of the scaled sums that are most correlated with the probabilities of R-loop formation in all transcription steps. The interactive 3D figures available online at `https://github.com/Arsuaga-Vazquez-Lab/RNA-Polynomial` show how the normalized overlapping sums change with respect to the transcriptional process of the RNA segments. We also observe in the interactive 3D figures that the values of normalized overlapping sums become relatively stable and the strong correlations appear at a very early stage of the transcriptional process, when the secondary structures of the segments have fewer than 50nt. The difference between the secondary structures with large coefficient sums and the ones with small coefficient sums are also captured by the tree-polynomial representations of the fewer-than-50nt-long secondary structures. This suggests that the fewer-than-50nt-long secondary structures already possess the feature for large coefficient sums, which potentially promotes R-loop formation.

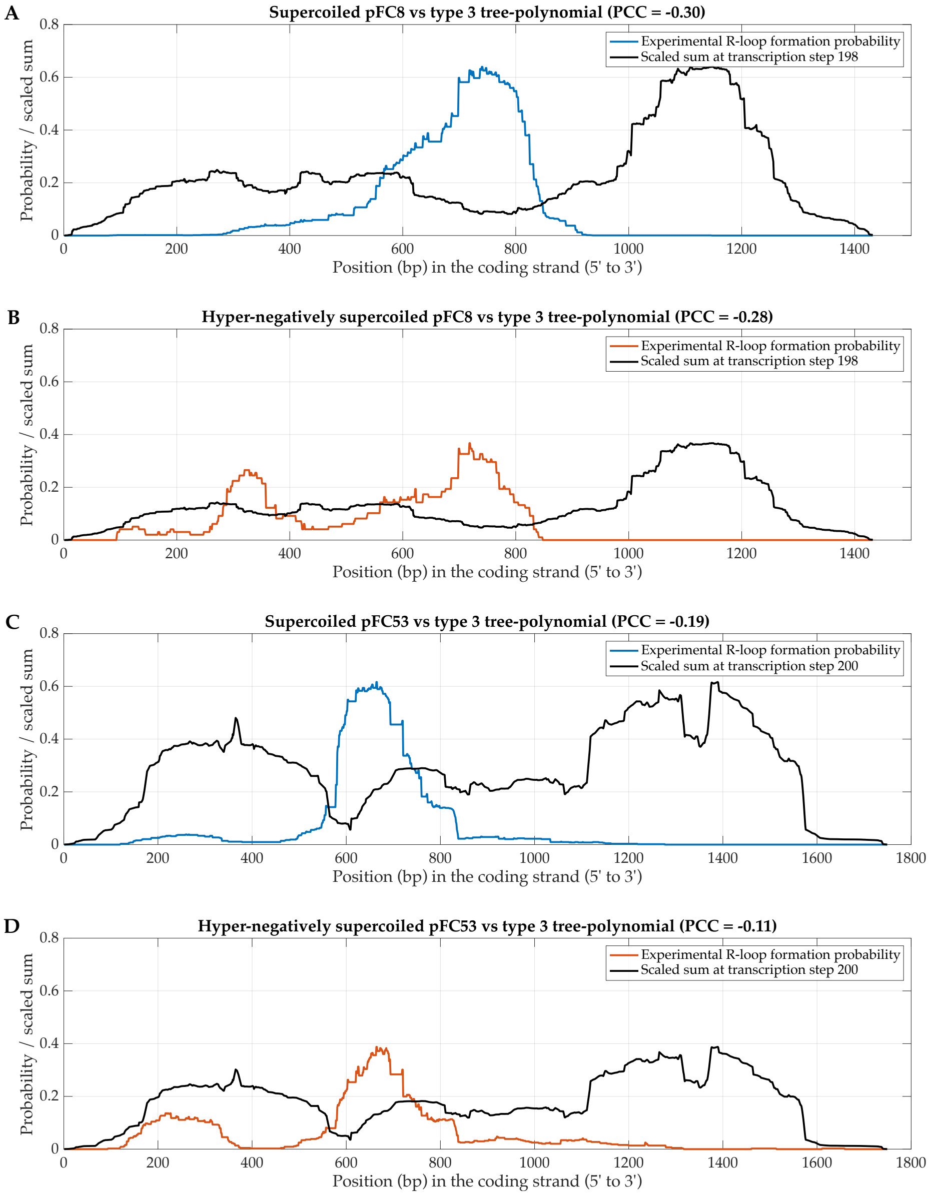

Fig D. The correlations between the type 3 scaled sums and the R-loop formation probability of pFC8 and pFC53 plasmids. The figure shows the correlations between the type 3 scaled sums (with the highest PCC in the last 10 transcription steps) and the R-loop formation probabilities of the supercoiled pFC8 plasmid (panel A) and of the hyper-negatively supercoiled pFC8 plasmid (panel B), and the correlations between the type 3 scaled sums (with the highest PCC in the last 10 transcription steps) and the R-loop formation probabilities of the supercoiled pFC53 plasmid (panel C) and of the hyper-negatively supercoiled pFC53 plasmid (panel D).

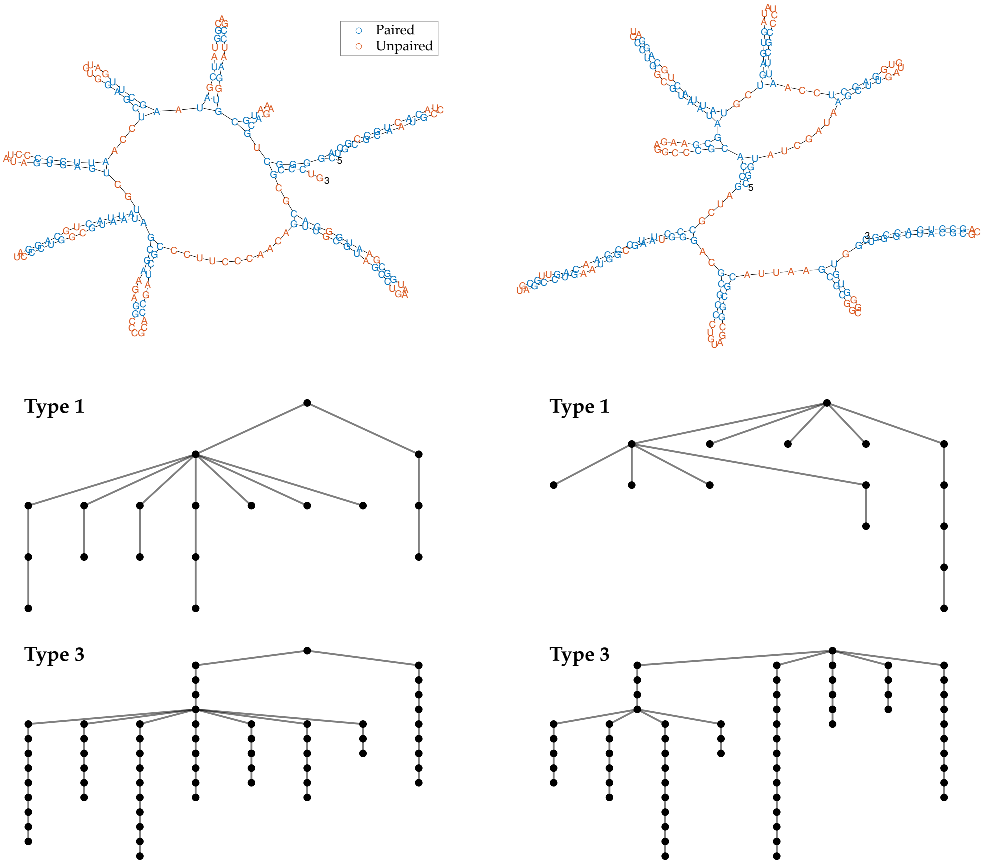

A pFC8 segment 1006 - 1205
Transcription step 198
Normalized sum (type 3): 1.00

B pFC8 segment 1057 - 1256
Transcription step 198
Normalized sum (type 3): 0.96

Fig E. Secondary structures of pFC8 RNA segments that have the largest type 3 coefficient sums. The figure shows the secondary structures of RNA segments of the pFC8 plasmid with the two largest type 3 coefficient sums at the transcription step with the highest PCC in the last 10 transcription steps. Type 1 and type 3 tree representations are displayed following the corresponding secondary structures.

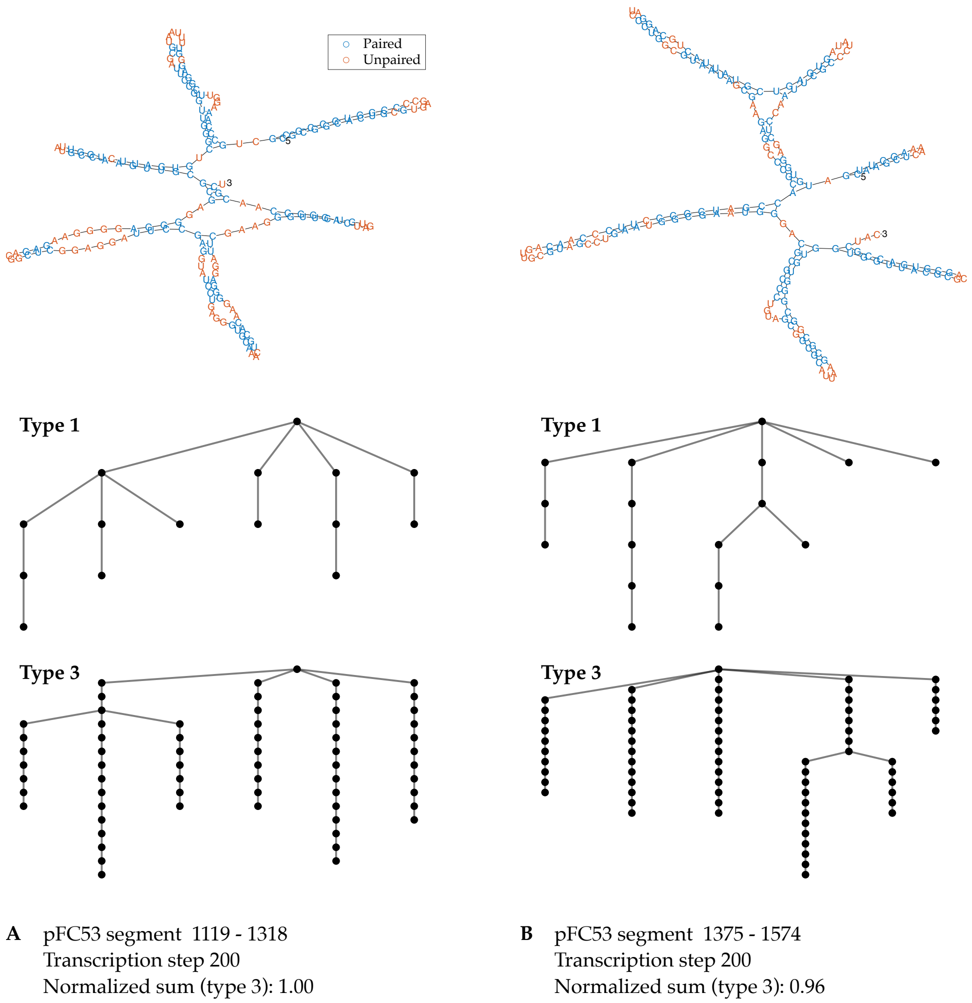

Fig F. Secondary structures of pFC53 RNA segments that have the largest type 3 coefficient sums. The figure shows the secondary structures of RNA segments of the pFC53 plasmid with the two largest type 3 coefficient sums at the transcription step with the highest PCC in the last 10 transcription steps. Type 1 and type 3 tree representations are displayed following the corresponding secondary structures.
