# Supplementary figures and images for: Tree polynomials identify a link between co-transcriptional R-loops and nascent RNA folding
Source: PLoS Comput Biol. 2024 Dec 13;20(12):e1012669. doi: 10.1371/journal.pcbi.1012669 (PMC11706388; doi:10.1371/journal.pcbi.1012669)

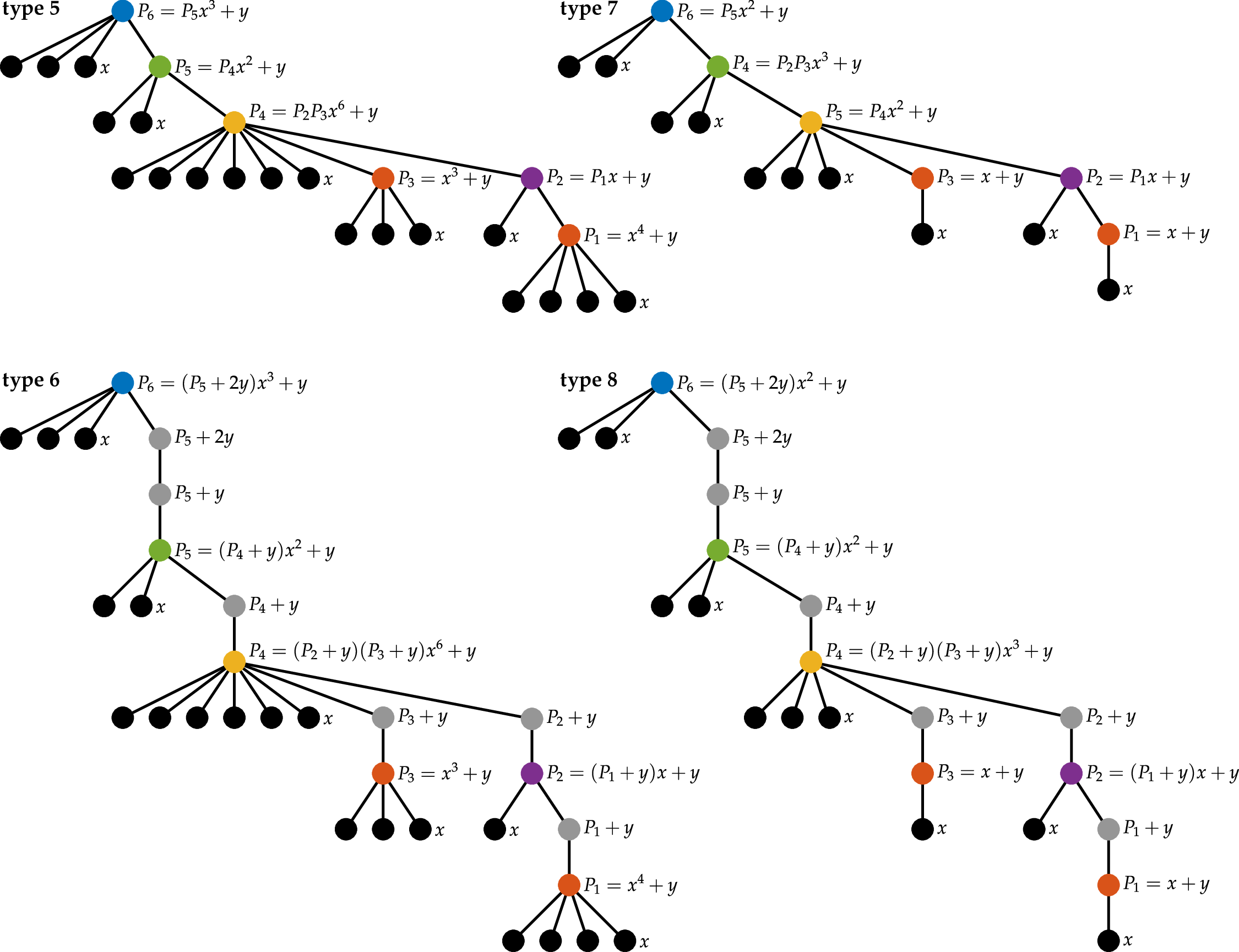

Supplement: S1 Fig — The figure shows the last four rooted tree representations and their corresponding polynomial representations of the RNA secondary structure displayed in Fig 1. Vertices in the trees are colored based on the loops or stem regions that they represent, and the black round vertices represent unpaired nucleotides. The leaf vertices in type 7 and type 8 tree representations represent artificial vertices introduced for grouping unpaired nucleotides. Alongside every tree representation, the recursive process of computing the corresponding polynomial from the leaf vertices to the root vertex is displayed. The polynomial at the root vertex of a rooted tree is the polynomial that represents the tree. (TIF) [file pcbi.1012669.s002.tif]

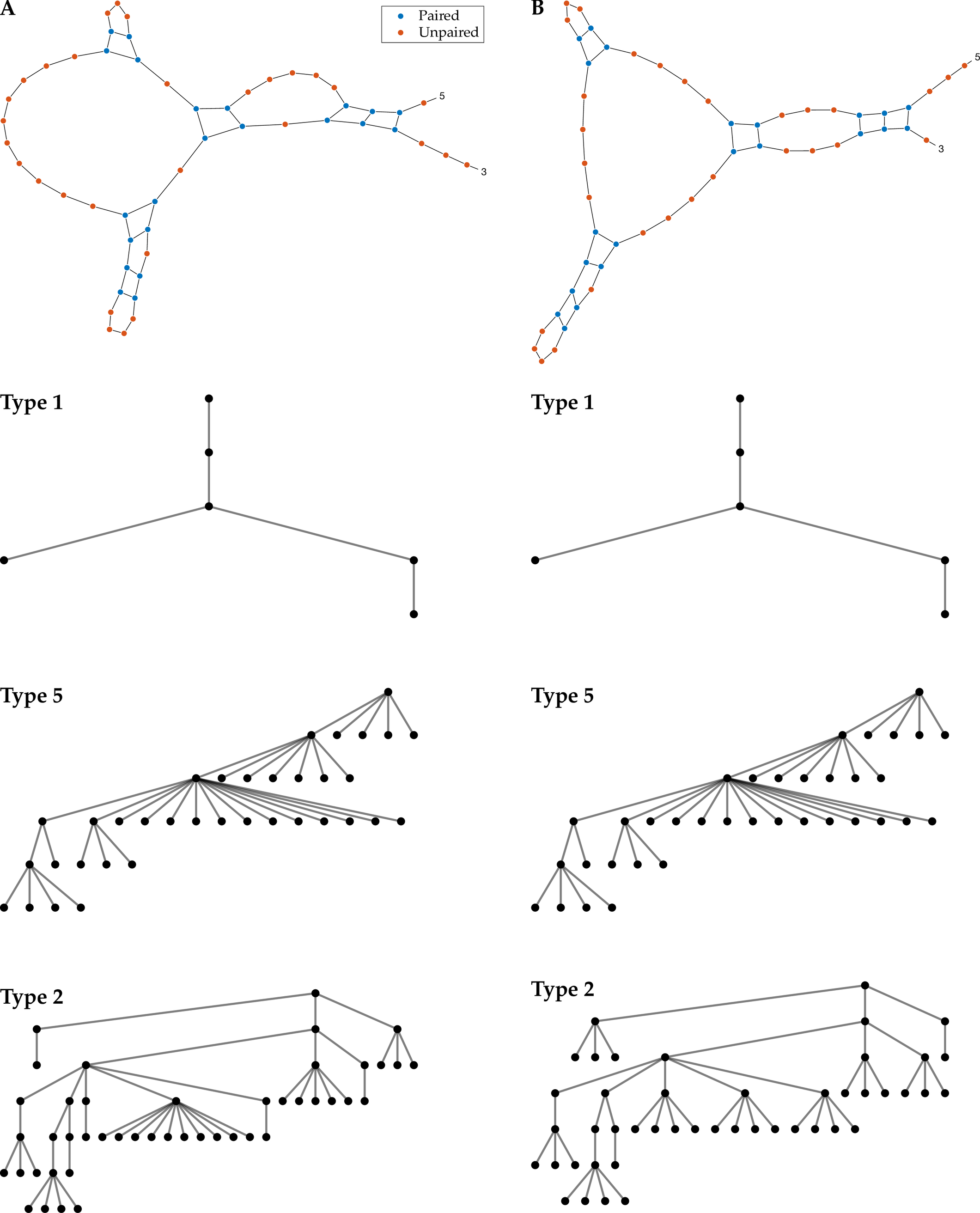

Supplement: S2 Fig — Panel A and panel B show a pair of 52nt long RNA secondary structures that are the same in loop-stem relation, loop size and stem size but different in loop group. Each panel also displays the corresponding type 1, type 5 and type 2 tree representations, where only type 2 tree representations distinguish between the RNA secondary structures. (TIF) [file pcbi.1012669.s003.tif]

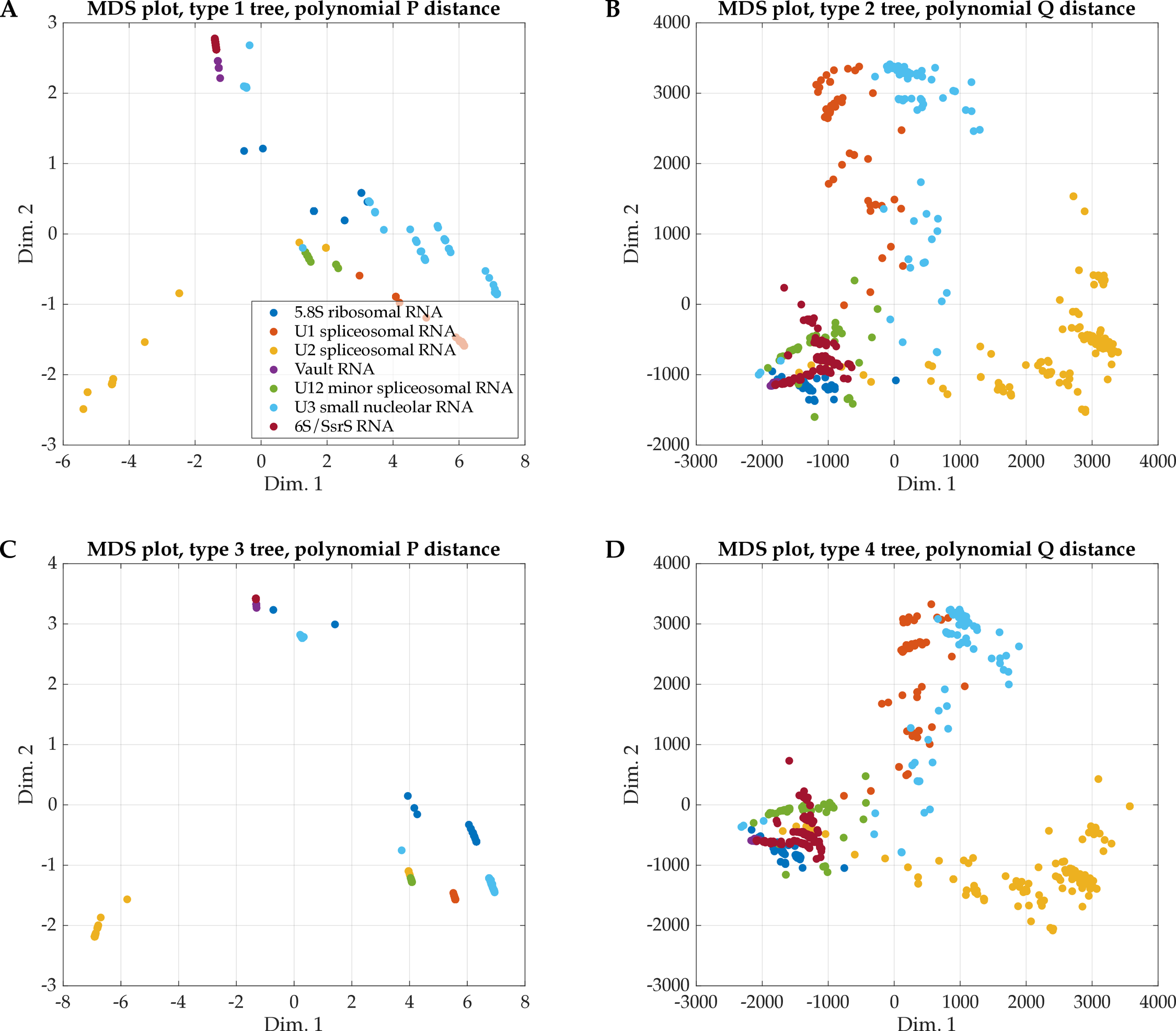

Supplement: S3 Fig — The top two panels show the MDS plots of the pairwise polynomial P distances between type 1 (panel A) and type 2 (panel B) tree-polynomial representations of the 735 ncRNA secondary structures in the bpRNA-Rfam-7 dataset. The bottom panels show the analogous MDS plots of the pairwise polynomial Q distances between type 3 (panel C) and type 4 (panel D) tree-polynomial representations. Each dot in a panel represents an ncRNA secondary structure of the ncRNA family corresponding to its color. We observe distinct clusters for each of the seven families of ncRNAs in all four plots. The plot for type 3 tree-polynomial representations corresponds to the best clustering results, with only two U3 small nucleolar RNAs (cyan) close to other clusters. (TIF) [file pcbi.1012669.s004.tif]

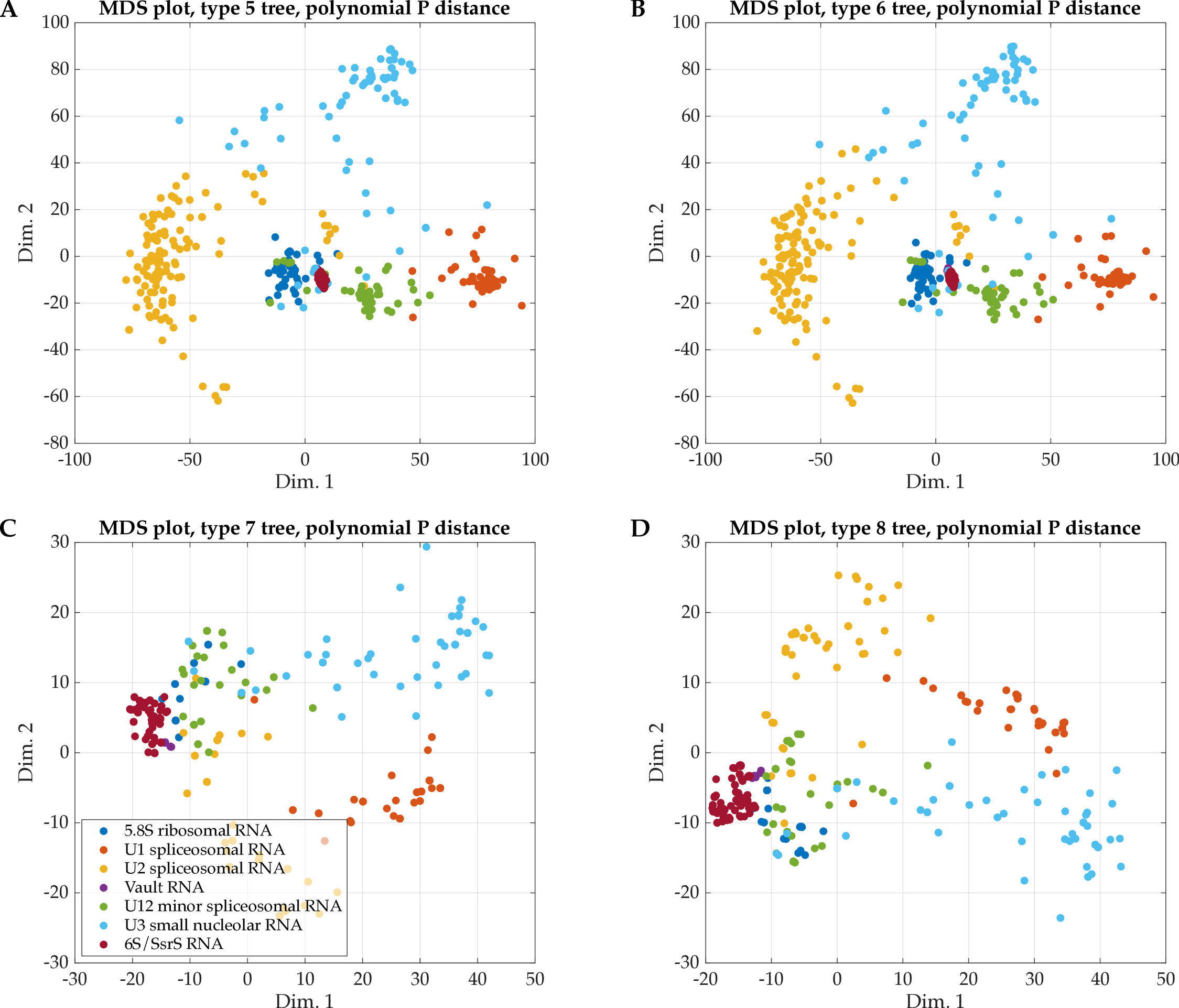

Supplement: S4 Fig — The figure shows the MDS plots of the pairwise polynomial P distances between type 5 (panel A) and type 6 (panel B) tree-polynomial representations of the 735 ncRNA secondary structures in the bpRNA-Rfam-7 dataset, and the MDS plots of the pairwise polynomial P distances between type 7 (panel C) and type 8 (panel D) tree-polynomial representations of the ncRNA secondary structures in the bpRNA-Rfam-7 dataset. Each dot in a panel represents an ncRNA secondary structure of the ncRNA family corresponding to its color. (TIF) [file pcbi.1012669.s005.tif]

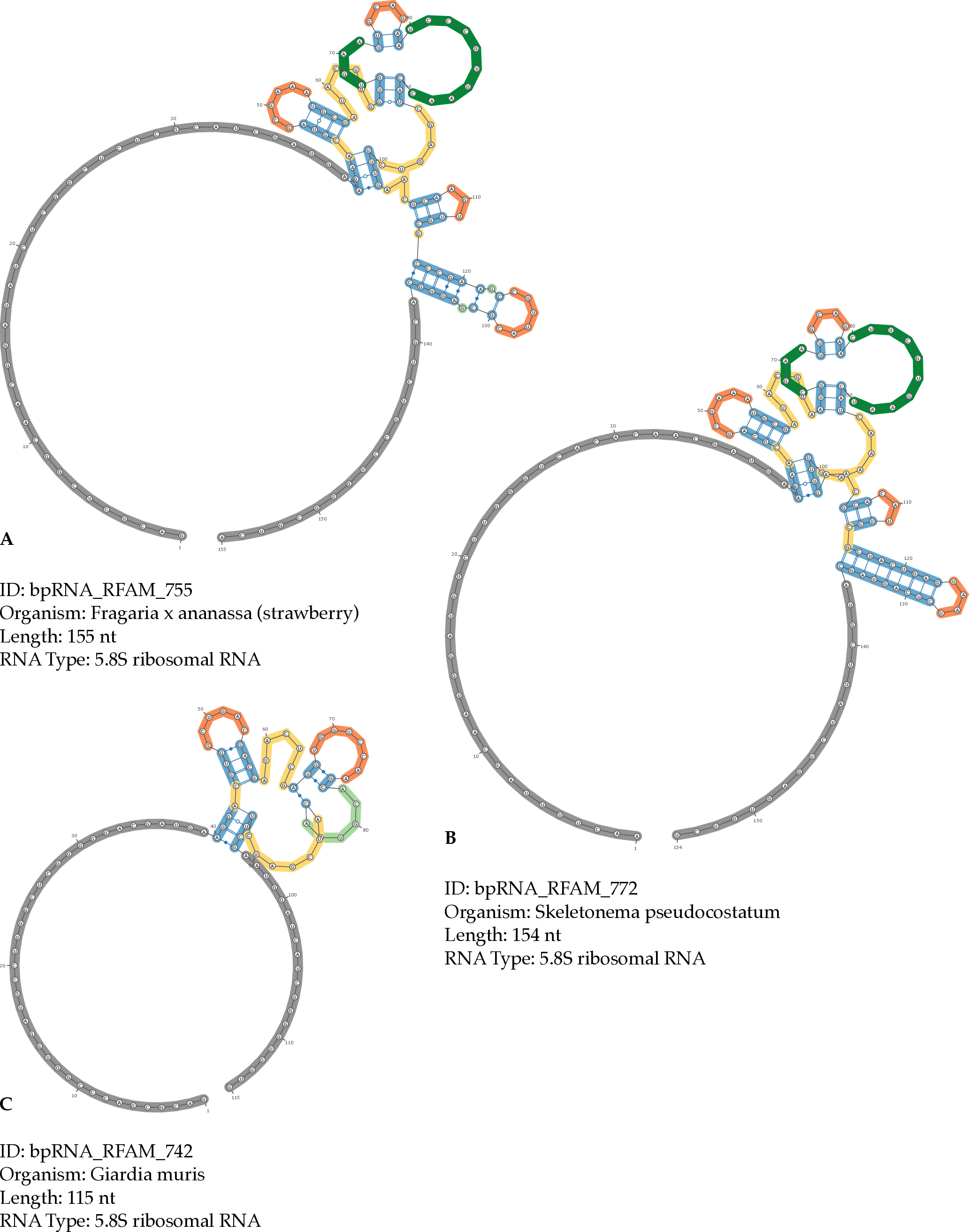

Supplement: S5 Fig — This figure shows examples of 5.8S ribosomal RNA secondary structures in the bpRNA-Rfam-7 dataset. We obtained the secondary structure diagrams and information from bpRNA-1m. (TIF) [file pcbi.1012669.s006.tif]

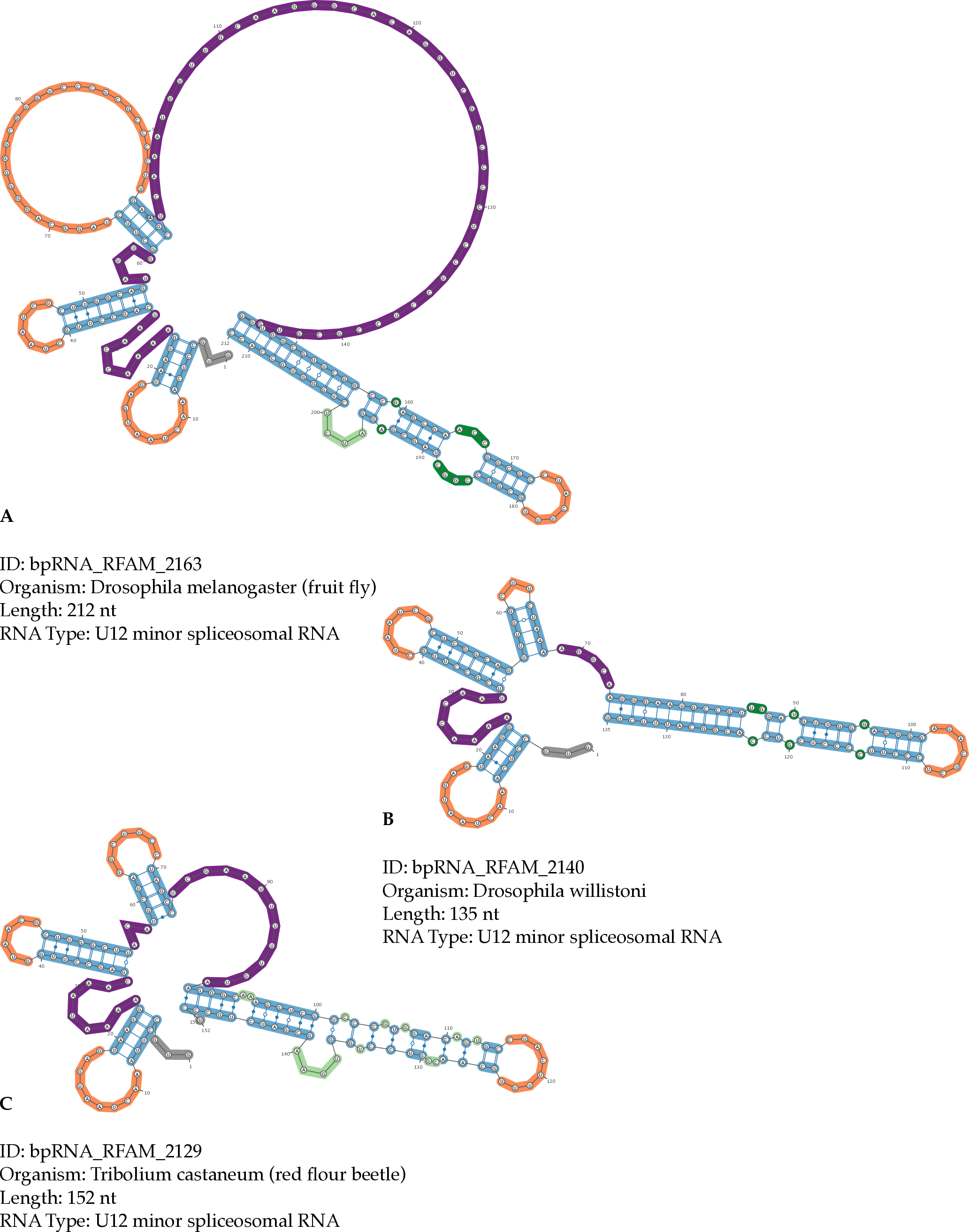

Supplement: S6 Fig — This figure shows examples of U12 minor spliceosomal RNA secondary structures in the bpRNA-Rfam-7 dataset. We obtained the secondary structure diagrams and information from bpRNA-1m. (TIF) [file pcbi.1012669.s007.tif]

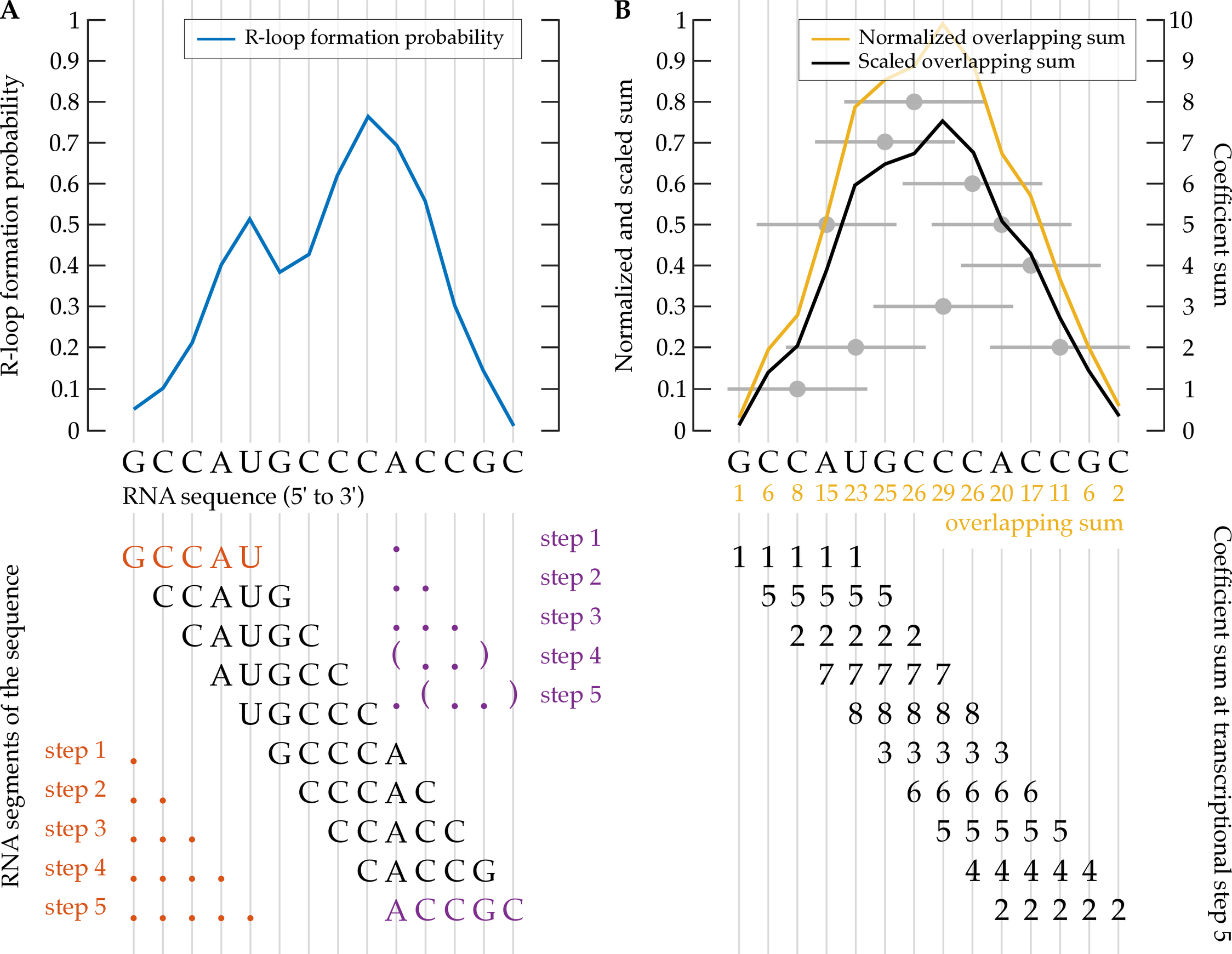

Supplement: S7 Fig — Panel A shows the probability of R-loop formation (blue) as a function of the RNA sequence, segments of the RNA sequence and the lists of RNA secondary structures (red and purple) of the first and the last RNA segments. Panel B shows the coefficient sums (black numbers) and the normalized sums (gray dots and horizontal lines) of the RNA segments at a transcription step and the process of computing the overlapping sum (yellow numbers below the sequence), normalized overlapping sum (yellow curve) and the scaled sum (black curve) of the RNA sequence from the coefficient sums. (TIF) [file pcbi.1012669.s008.tif]

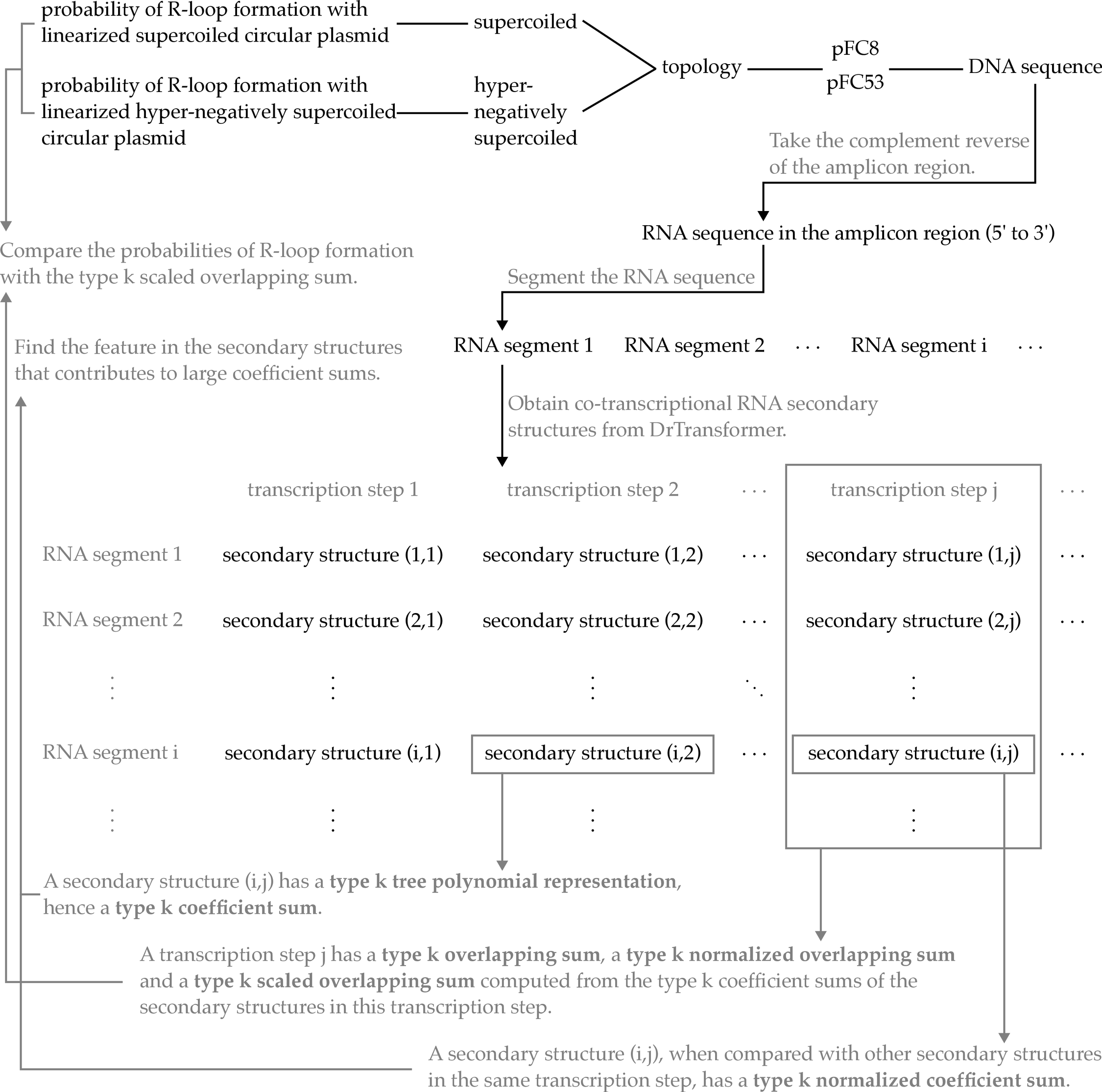

Supplement: S8 Fig — This diagram shows how we process the DNA sequence data of the two plasmids and compare the obtained RNA secondary structure with the probabilities of R-loop formation. (TIF) [file pcbi.1012669.s009.tif]

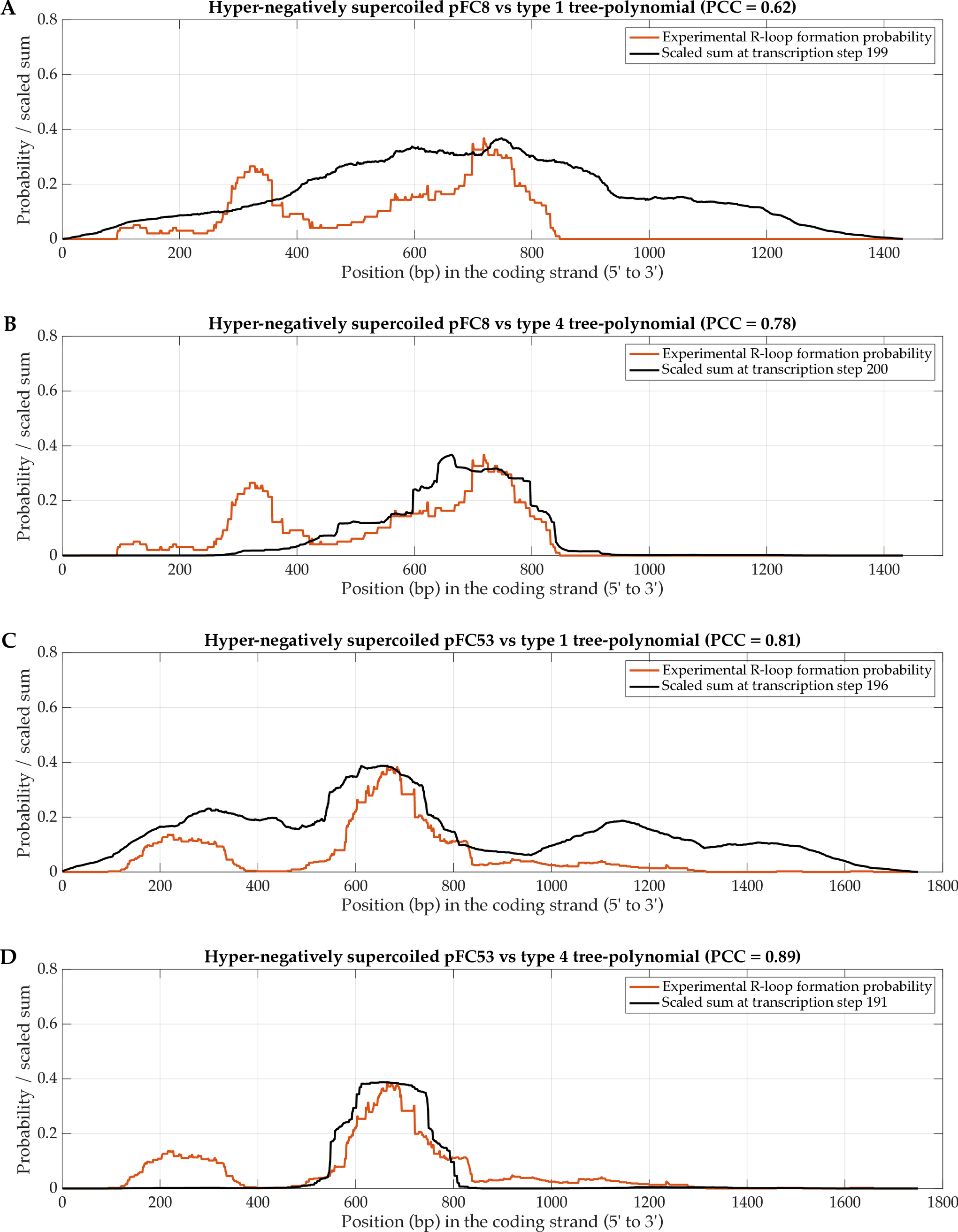

Supplement: S9 Fig — The figure shows the experimental probability of R-loop formation for the hyper-negatively supercoiled pFC8 plasmid with the type 1 (panel A) and the type 4 (panel B) scaled sums, and the experimental probability of R-loop formation for the hyper-negatively supercoiled pFC53 plasmid with the type 1 (panel C) and the type 4 (panel D) scaled sums. The experimental probabilities of R-loop formation are from [6]. The displayed scaled sums have the highest PCC in the last 10 transcription steps. (TIF) [file pcbi.1012669.s010.tif]

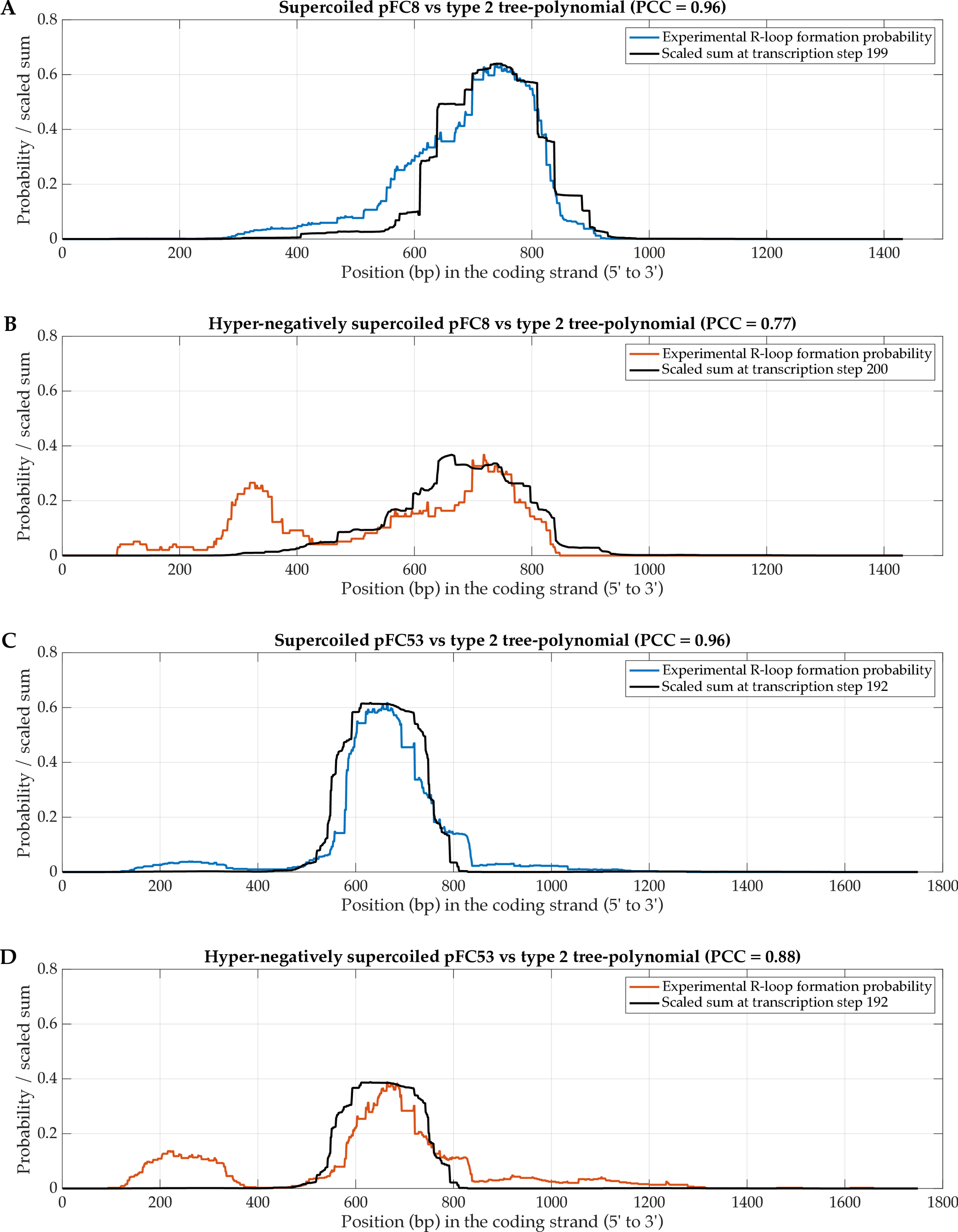

Supplement: S10 Fig — The figure shows the correlations between the type 2 scaled sums (with the highest PCC in the last 10 transcription steps) and the R-loop formation probabilities of the supercoiled pFC8 plasmid (panel A) and of the hyper-negatively supercoiled pFC8 plasmid (panel B), and the correlations between the type 2 scaled sums (with the highest PCC in the last 10 transcription steps) and the R-loop formation probabilities of the supercoiled pFC53 plasmid (panel C) and of the hyper-negatively supercoiled pFC53 plasmid (panel D). (TIF) [file pcbi.1012669.s011.tif]

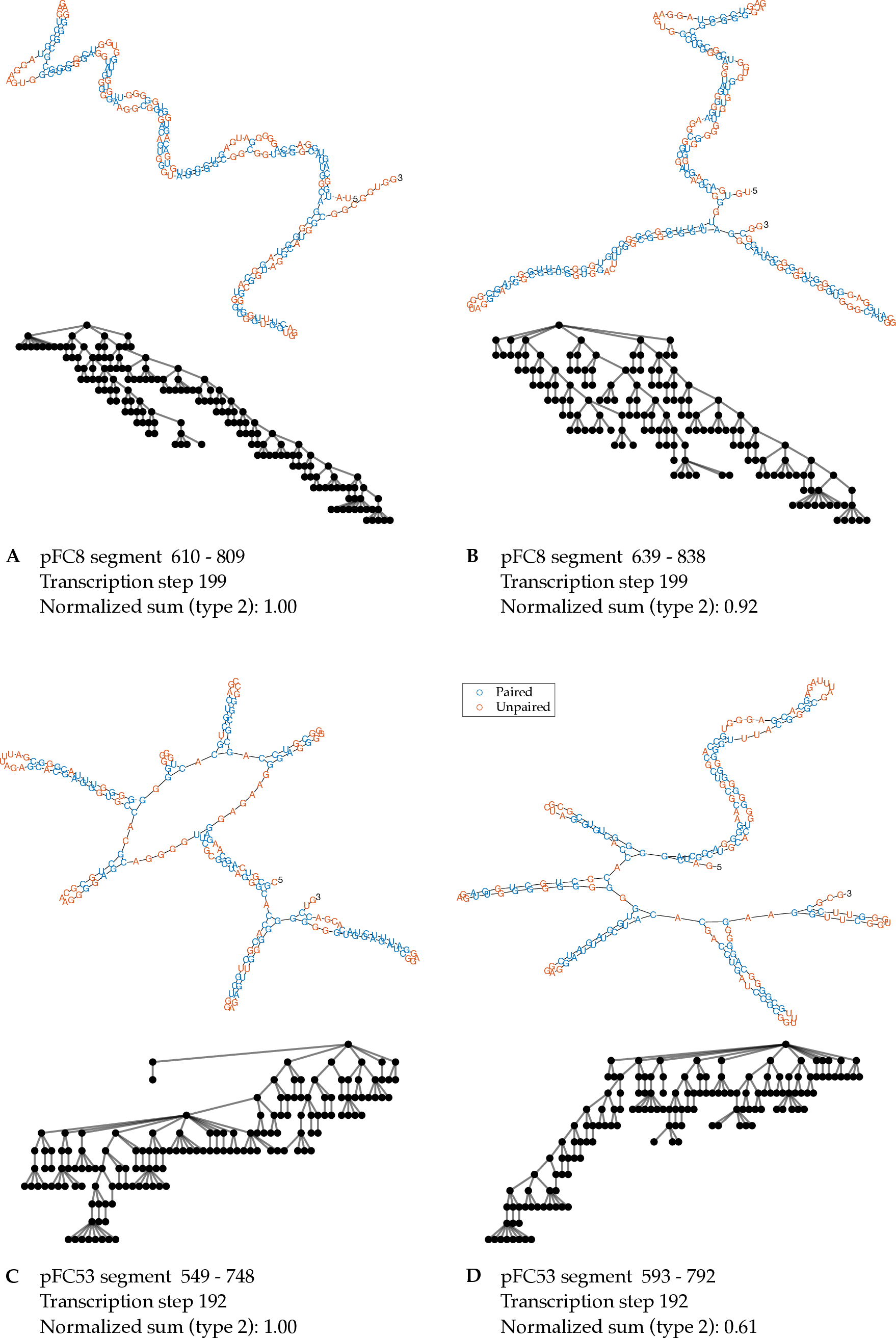

Supplement: S11 Fig — The figure shows the secondary structures of RNA segments of the pFC8 plasmid (panel A and B) and the pFC53 plasmid (panel C and D) with the two largest type 2 coefficient sums at the transcription step with the highest PCC in the last 10 transcription steps. Type 2 tree representations are displayed following the corresponding secondary structures. (TIF) [file pcbi.1012669.s012.tif]

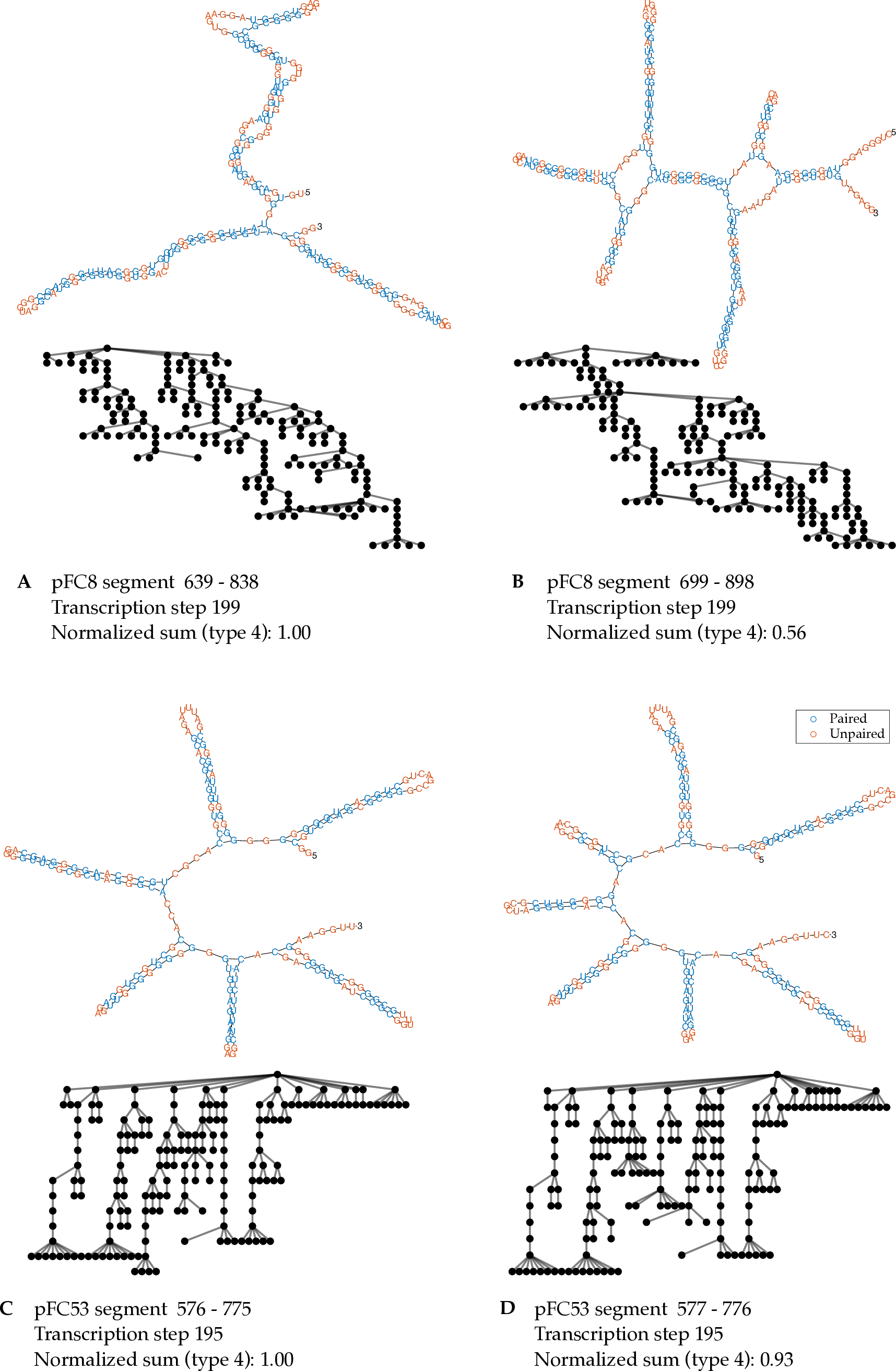

Supplement: S12 Fig — The figure shows the secondary structures of RNA segments of the pFC8 plasmid (panel A and B) and the pFC53 plasmid (panel C and D) with the two largest type 4 coefficient sums at the transcription step with the highest PCC in the last 10 transcription steps. Type 4 tree representations are displayed following the corresponding secondary structures. (TIF) [file pcbi.1012669.s013.tif]

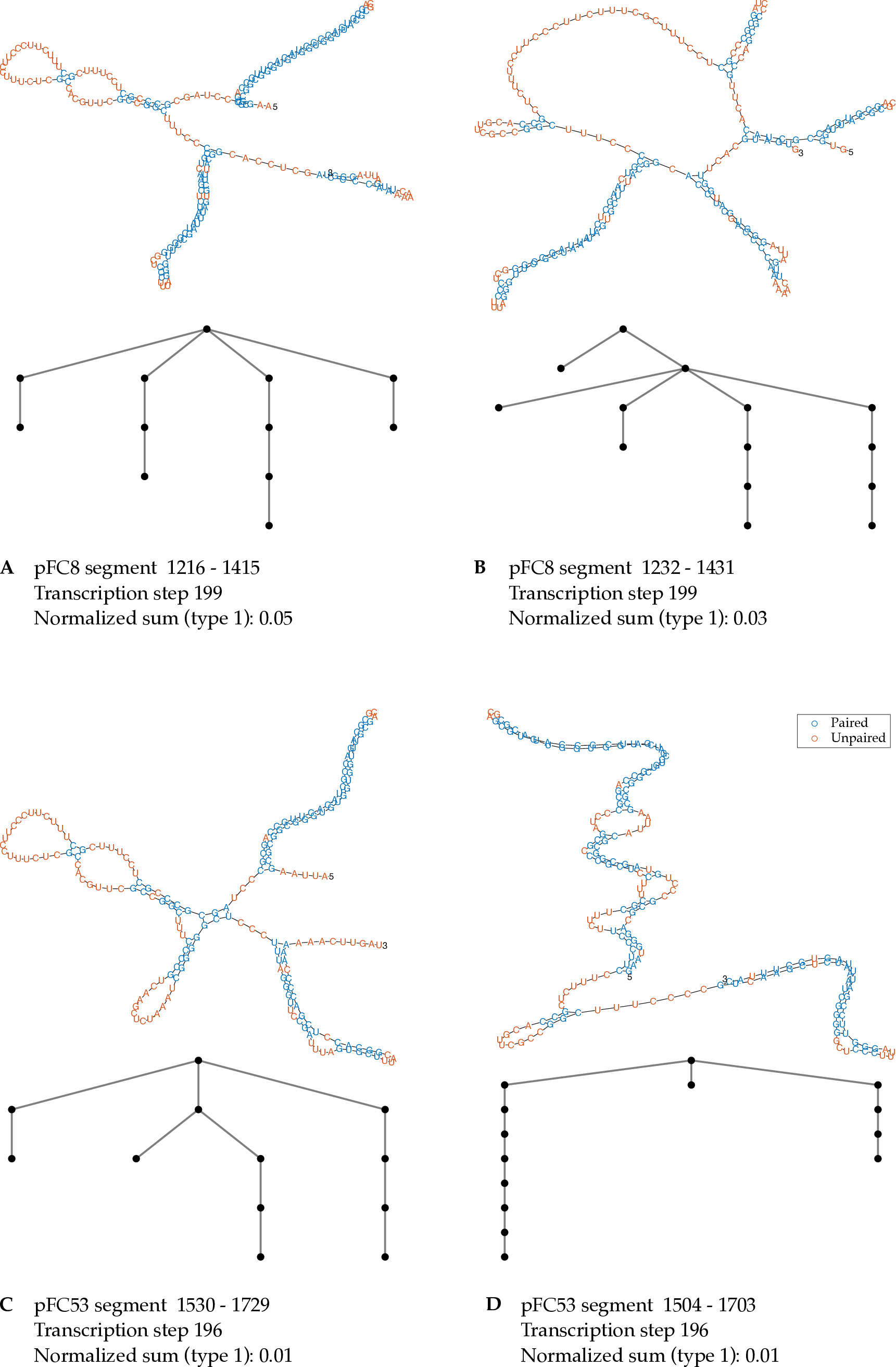

Supplement: S13 Fig — The figure shows the secondary structures of RNA segments of the pFC8 plasmid (panel A and B) and the pFC53 plasmid (panel C and D) near the 3’ end of the amplicon region that have the two largest type 1 coefficient sums at the transcription step with the highest PCC in the last 10 transcription steps. Type 1 tree representations are displayed following the corresponding secondary structures. (TIF) [file pcbi.1012669.s014.tif]

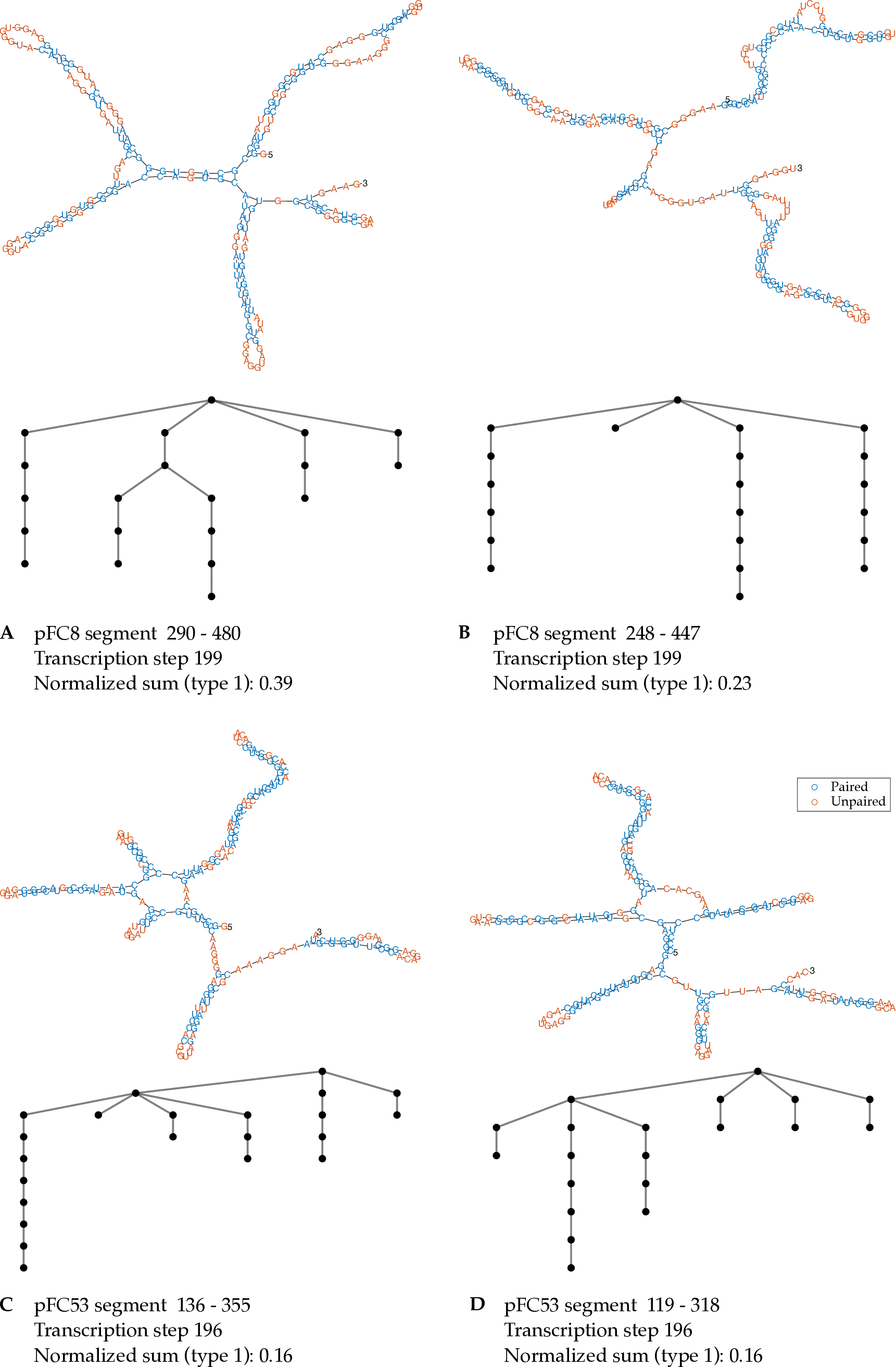

Supplement: S14 Fig — The figure shows the secondary structures of RNA segments of the pFC8 plasmid (panel A and B) and the pFC53 plasmid (panel C and D) in the minor peak of R-loop formation that have the two largest type 1 coefficient sums at the transcription step with the highest PCC in the last 10 transcription steps. Type 1 tree representations are displayed following the corresponding secondary structures. (TIF) [file pcbi.1012669.s015.tif]
