# Supplementary material for: Tree polynomials identify a link between co-transcriptional R-loops and nascent RNA folding
Source: PLoS Comput Biol. 2024 Dec 13;20(12):e1012669. doi: 10.1371/journal.pcbi.1012669 (PMC11706388; doi:10.1371/journal.pcbi.1012669)
Supplement: S1 Table — The first column shows the seven Rfam ncRNA families selected in the study. The second column shows the number of RNA secondary structures without pseudoknots in each family. The third column shows the average length of the ncRNA sequences in each family. (PDF) [file pcbi.1012669.s016.pdf]

Non-coding RNA family	Number of secondary structures	Average length
5.8S ribosomal RNA	61	152.21 nt
U1 spliceosomal RNA	100	162.25 nt
U2 spliceosomal RNA	208	190.51 nt
Vault RNA	73	100.93 nt
U12 minor spliceosomal RNA	62	161.16 nt
U3 small nucleolar RNA	82	208.29 nt
6S/SsrS RNA	149	180.01 nt
Total/Average	735	171.97 nt

S1 Table. The bpRNA-Rfam-7 dataset of non-coding RNA (ncRNA) secondary structures. The first column shows the seven Rfam ncRNA families selected in the study. The second column shows the number of RNA secondary structures without pseudoknots in each family. The third column shows the average length of the ncRNA sequences in each family.
