# Supplementary material for: Tree polynomials identify a link between co-transcriptional R-loops and nascent RNA folding
Source: PLoS Comput Biol. 2024 Dec 13;20(12):e1012669. doi: 10.1371/journal.pcbi.1012669 (PMC11706388; doi:10.1371/journal.pcbi.1012669)
Supplement: S2 Table — The table shows the bpRNA ID of the secondary structures from the seven ncRNA families in the Rfam dataset. The asterisk superscript indicates that there are five U3 small nucleolar RNA secondary structures (with ID 2946, 2951, 2965, 2970 and 2998) that have pseudoknots and are not included in the bpRNA-Rfam-7 dataset. (PDF) [file pcbi.1012669.s017.pdf]

Non-coding RNA family	bpRNA ID
5.8S ribosomal RNA	bpRNA_RFAM_713 to bpRNA_RFAM_773
U1 spliceosomal RNA	bpRNA_RFAM_774 to bpRNA_RFAM_873
U2 spliceosomal RNA	bpRNA_RFAM_874 to bpRNA_RFAM_1801
Vault RNA	bpRNA_RFAM_2036 to bpRNA_RFAM_2108
U12 minor spliceosomal RNA	bpRNA_RFAM_2109 to bpRNA_RFAM_2170
U3 small nucleolar RNA	bpRNA_RFAM_2941 to bpRNA_RFAM_3027*
6S/SsrS RNA	bpRNA_RFAM_3028 to bpRNA_RFAM_3176

S2 Table. The bpRNA IDs of ncRNA secondary structures in the bpRNA-Rfam-7 dataset. The table shows the bpRNA ID of the secondary structures from the seven ncRNA families in the Rfam dataset. The asterisk superscript indicates that there are five U3 small nucleolar RNA secondary structures (with ID 2946, 2951, 2965, 2970 and 2998) that have pseudoknots and are not included in the bpRNA-Rfam-7 dataset.
