# Supplementary material for: Tree polynomials identify a link between co-transcriptional R-loops and nascent RNA folding
Source: PLoS Comput Biol. 2024 Dec 13;20(12):e1012669. doi: 10.1371/journal.pcbi.1012669 (PMC11706388; doi:10.1371/journal.pcbi.1012669)
Supplement: S3 Table — This table shows the mean misclassification rates (MMRs) of clustering all 16,039 Rfam RNA secondary structures with length between 100nt and 200nt and without pseudoknots in the bpRNA-1m database. (PDF) [file pcbi.1012669.s018.pdf]

Type	type 1	type 2	type 3	type 4	type 5	type 6	type 7	type 8
MMR	6.22%	16.06%	2.97%	16.34%	8.93%	9.05%	23.60%	22.82%

S3 Table. Mean misclassification rates of clustering RNA secondary structures in the bpRNA-Rfam-large dataset. This table shows the mean misclassification rates (MMRs) of clustering all 16,039 Rfam RNA secondary structures with length between 100nt and 200nt and without pseudoknots in the bpRNA-1m database.
