# Supplementary material for: Tree polynomials identify a link between co-transcriptional R-loops and nascent RNA folding
Source: PLoS Comput Biol. 2024 Dec 13;20(12):e1012669. doi: 10.1371/journal.pcbi.1012669 (PMC11706388; doi:10.1371/journal.pcbi.1012669)
Supplement: S4 Table — The first columns show the types of tree-polynomial representations. The last four columns show the highest Pearson’s correlation coefficients (PCCs) between the scaled sums of the tree-polynomial representations and the probabilities of R-loop formation of the corresponding plasmids in the last 10 transcription steps. Inside the parentheses after the PCCs are the transcription steps of the corresponding scaled sums. (PDF) [file pcbi.1012669.s019.pdf]

Type	pFC8 supercoiled	pFC8 hyper-negatively supercoiled	pFC53 supercoiled	pFC53 hyper-negatively supercoiled
1	0.77 (step 199)	0.62 (step 199)	0.79 (step 196)	0.81 (step 196)
2	0.96 (step 199)	0.77 (step 200)	0.96 (step 192)	0.88 (step 192)
3	-0.30 (step 198)	-0.28 (step 198)	-0.19 (step 200)	-0.11 (step 200)
4	0.97 (step 199)	0.78 (step 200)	0.97 (step 195)	0.89 (step 191)
5	0.60 (step 197)	0.48 (step 197)	0.64 (step 195)	0.66 (step 199)
6	-0.31 (step 198)	-0.34 (step 198)	-0.21 (step 200)	-0.18 (step 194)
7	0.60 (step 197)	0.48 (step 197)	0.64 (step 195)	0.66 (step 199)
8	-0.31 (step 198)	-0.34 (step 198)	-0.21 (step 200)	-0.18 (step 194)

S4 Table. The highest Pearson's correlation coefficients between the scaled sums and the probabilities of R-loop formation of the last 10 transcription steps. The first columns show the types of tree-polynomial representations. The last four columns show the highest Pearson's correlation coefficients (PCCs) between the scaled sums of the tree-polynomial representations and the probabilities of R-loop formation of the corresponding plasmids in the last 10 transcription steps. Inside the parentheses after the PCCs are the transcription steps of the corresponding scaled sums.
