# Supplementary material for: Tree polynomials identify a link between co-transcriptional R-loops and nascent RNA folding
Source: PLoS Comput Biol. 2024 Dec 13;20(12):e1012669. doi: 10.1371/journal.pcbi.1012669 (PMC11706388; doi:10.1371/journal.pcbi.1012669)
Supplement: S5 Table — The first columns show the types of tree-polynomial representations. The last four columns show the highest Pearson’s correlation coefficients (PCCs) between the scaled sums of the tree-polynomial representations and the probabilities of R-loop formation of the corresponding plasmids over all transcription steps. Inside the parentheses after the PCCs are the transcription steps of the corresponding scaled sums. (PDF) [file pcbi.1012669.s020.pdf]

Type	pFC8 supercoiled	pFC8 hyper-negatively supercoiled	pFC53 supercoiled	pFC53 hyper-negatively supercoiled
1	0.77 (step 199)	0.62 (step 181)	0.79 (step 196)	0.81 (step 196)
2	0.96 (step 199)	0.77 (step 200)	0.97 (step 134)	0.90 (step 121)
3	0.32 (step 20)	0.42 (step 16)	-0.21 (step 167)	0.27 (step 9)
4	0.97 (step 199)	0.78 (step 200)	0.97 (step 150)	0.90 (step 153)
5	0.62 (step 168)	0.49 (step 181)	0.71 (step 147)	0.72 (step 156)
6	0.32 (step 20)	0.39 (step 16)	0.24 (step 67)	0.27 (step 9)
7	0.62 (step 168)	0.49 (step 181)	0.71 (step 147)	0.72 (step 156)
8	0.32 (step 20)	0.39 (step 16)	0.24 (step 67)	0.27 (step 9)

S5 Table. The highest Pearson's correlation coefficients between the scaled sums and the probabilities of R-loop formation in all transcription steps. The first columns show the types of tree-polynomial representations. The last four columns show the highest Pearson's correlation coefficients (PCCs) between the scaled sums of the tree-polynomial representations and the probabilities of R-loop formation of the corresponding plasmids over all transcription steps. Inside the parentheses after the PCCs are the transcription steps of the corresponding scaled sums.
